# Covariance properties under natural image transformations for the generalised Gaussian derivative model for visual receptive fields

**DOI:** 10.3389/fncom.2023.1189949

**Published:** 2023-06-15

**Authors:** Tony Lindeberg

**Affiliations:** Computational Brain Science Lab, Division of Computational Science and Technology, KTH Royal Institute of Technology, Stockholm, Sweden

**Keywords:** receptive field, Image transformations, scale covariance, affine covariance, Galilean covariance, primary visual cortex, vision, theoretical neuroscience

## Abstract

The property of covariance, also referred to as equivariance, means that an image operator is well-behaved under image transformations, in the sense that the result of applying the image operator to a transformed input image gives essentially a similar result as applying the same image transformation to the output of applying the image operator to the original image. This paper presents a theory of geometric covariance properties in vision, developed for a generalised Gaussian derivative model of receptive fields in the primary visual cortex and the lateral geniculate nucleus, which, in turn, enable geometric invariance properties at higher levels in the visual hierarchy. It is shown how the studied generalised Gaussian derivative model for visual receptive fields obeys true covariance properties under spatial scaling transformations, spatial affine transformations, Galilean transformations and temporal scaling transformations. These covariance properties imply that a vision system, based on image and video measurements in terms of the receptive fields according to the generalised Gaussian derivative model, can, to first order of approximation, handle the image and video deformations between multiple views of objects delimited by smooth surfaces, as well as between multiple views of spatio-temporal events, under varying relative motions between the objects and events in the world and the observer. We conclude by describing implications of the presented theory for biological vision, regarding connections between the variabilities of the shapes of biological visual receptive fields and the variabilities of spatial and spatio-temporal image structures under natural image transformations. Specifically, we formulate experimentally testable biological hypotheses as well as needs for measuring population statistics of receptive field characteristics, originating from predictions from the presented theory, concerning the extent to which the shapes of the biological receptive fields in the primary visual cortex span the variabilities of spatial and spatio-temporal image structures induced by natural image transformations, based on geometric covariance properties.

## 1. Introduction

**Figure 1:**
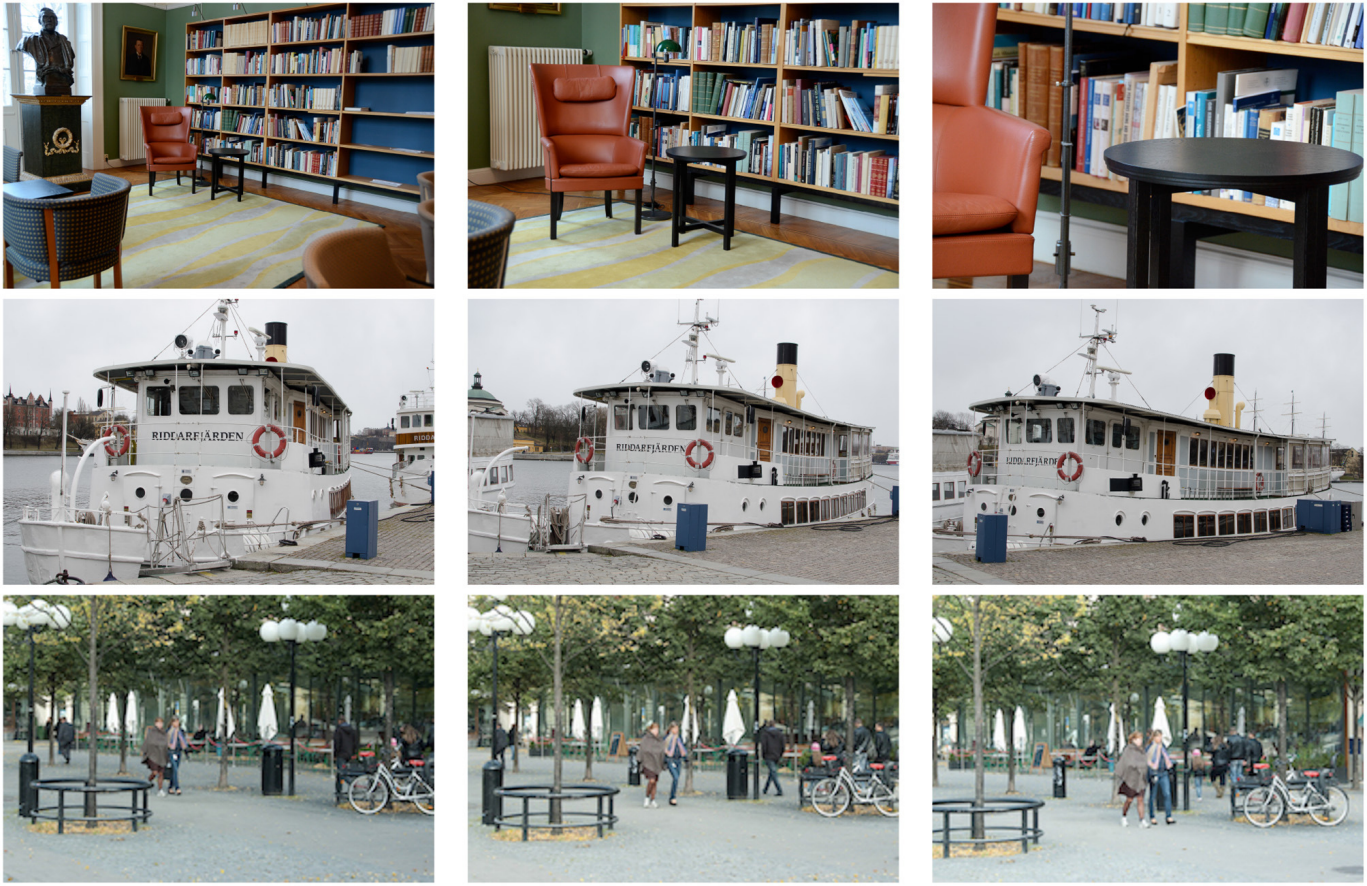
Due to the effects of natural image transformations, the perspective projections of spatial objects and spatio-temporal events in the world may appear in substantially different ways depending on the viewing conditions. This figure shows images from natural scenes under variations in (top row) the viewing distance, (middle row) the viewing direction and (bottom row) the relative motion between objects in the world and the observer. By approximating the non-linear perspective mapping by a local linearization (the derivative), these geometric image transformations can, to first order of approximation, be modelled by spatial scaling transformations, spatial affine transformations and Galilean transformations.

The image and video data, that a vision system is exposed to, is subject to geometric image transformations, due to variations in the viewing distance, the viewing direction and the relative motion of objects in the world relative to the observer. These natural image transformations do, in turn, cause a substantial variability, by which an object or event in the world may appear in different ways to a visual observer:

- Variations in the distance between objects in the world and the observer lead to variations in scale, often up to over several orders of magnitude, which, to first order of approximation of the perspective mapping, can be modelled as (uniform) spatial scaling transformations (see Figure 1 top row).
- Variations in the viewing direction between the object and the observer will lead to a wider class of local image deformations, with different amount of foreshortening in different spatial directions, which, to first order of approximation, can be modelled as local spatial affine transformations, where the monocular foreshortening will depend upon the slant angle of a surface patch, and correspond to different amount of scaling in different directions, also complemented by a skewing transformations (see Figure 1 middle row).
- Variations in the relative motion between objects in the world and the viewing direction will additionally transform the joint spatio-temporal video domain in a way that, to first order of approximation, can be modelled as local Galilean transformations. (see Figure 1 bottom row).

In this paper, we will study the transformation properties of receptive field^1^ responses in the primary visual cortex (V1) under these classes of geometric image transformations, as well as for temporal scaling transformations that correspond to objects in the world that move as well as spatio-temporal events that occur faster or slower. We will also study the transformation properties of neurons in the lateral geniculate nucleus (LGN) under a lower variability over spatial scaling transformations, spatial rotations, temporal scaling transformations and Galilean transformations. An overall message that we will propose is that if the family of visual receptive fields is covariant (or equivariant^2^) under these classes of natural image transformations, as the family of generalised Gaussian derivative based receptive fields that we will study is, then these covariance properties make it possible for the vision system to, up to first order of approximation, match the receptive field responses under the huge variability caused by these geometric image transformations, which, in turn, will make it possible for the vision system to infer more accurate cues to the structure of the environment.

We will then use these theoretical results to express predictions concerning biological receptive fields in the primary visual cortex and the lateral geniculate nucleus, to characterize to what extent the variability of receptive field shapes, in terms of receptive fields tuned to different orientations, motion directions as well as spatial and temporal scales, as has been found by neurophysiological cell recordings of biological neurons (DeAngelis et al., 1995; Ringach, 2002, 2004; DeAngelis and Anzai, 2004; Conway and Livingstone, 2006; Johnson et al., 2008; De and Horwitz, 2021), could be explained by the primary visual cortex computing a covariant image representation over the basic classes of natural geometric image transformations.

### 1.1. The importance of covariant receptive fields to handle the influence of natural image transformations

For spatial receptive fields, that integrate spatial information over non-infinitesimal regions over image space, the specific way that the receptive fields accumulate evidence over these non-infinitesimal support regions will crucially determine how well they are able to handle the huge variability of visual stimuli caused by geometric image transformations.

**Figure 2:**
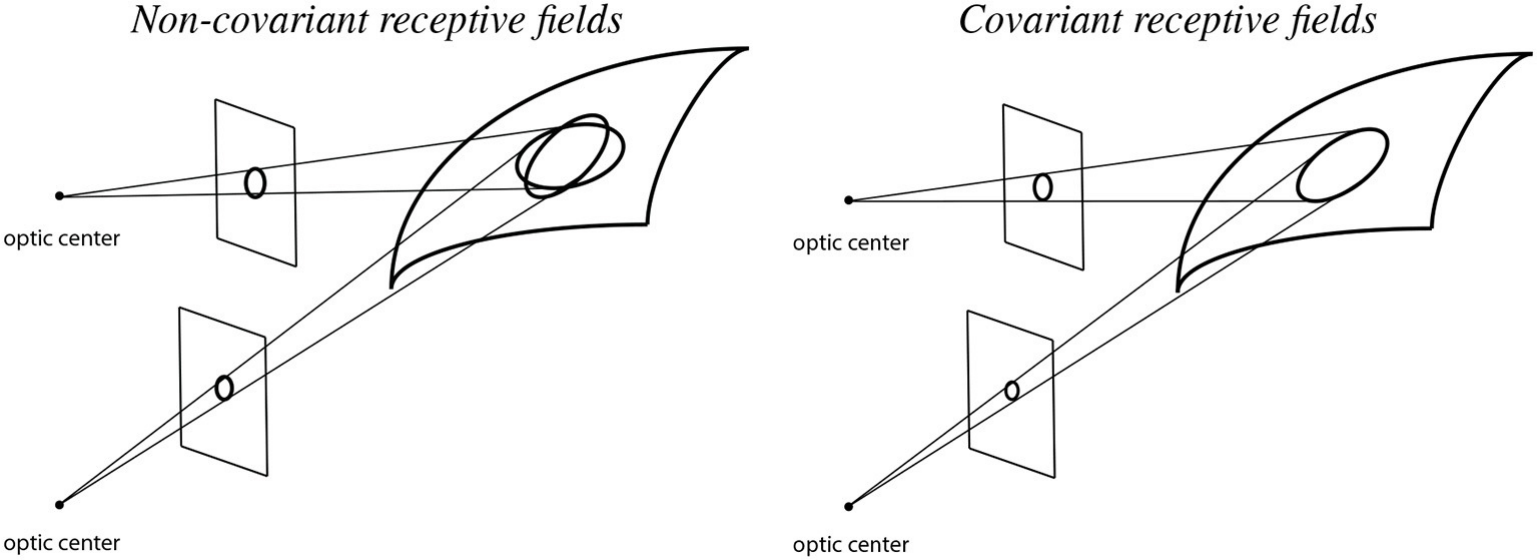
Illustration of backprojected receptive fields for a visual observer that observes a smooth surface from two different viewing directions in the cases of **(Left)** non-covariant receptive fields and **(Right)** covariant receptive fields. When using non-covariant receptive fields, the backprojected receptive fields will not match, which may cause problems for higher level visual processes that aim at computing estimates of local surface orientation, whereas when using covariant receptive fields, the backprojected receptive fields can be adjusted to match, which in turn enable more accurate estimates of local surface orientation (See the text in Section 1.1. for a more detailed explanation.).

To illustrate the importance of natural image transformations with respect to image measurements in terms of receptive fields, consider a visual observer that views a 3-D scene from two different viewing directions as shown in Figure 2.

Let us initially assume that the receptive fields for the two different viewing conditions are based on image measurements over rotationally symmetric support regions in the two image domains only. (More precisely, with respect to the theory that is to be developed next, concerning receptive fields that are defined from spatial derivatives of spatial smoothing kernels, the first crucial factor in this model concerns the support regions of the underlying spatial smoothing kernels of the spatial receptive fields in the two image domains).

If we backproject these rotationally symmetric support regions in the two image domains to the tangent plane of the surface in the world, then these backprojections will, to first order of approximation, be ellipses. Those ellipses will, however, not coincide in the tangent plane of the surface, implying that if we try to make use of the difference between the image measurements over the two spatial image domains, then there will be an unavoidable source of error, caused by the difference between the backprojected receptive fields, which will substantially affect the accuracy when computing cues to the structure of the world.

If we instead allow ourselves to consider different shapes of the support regions in the two image domains, and also match the parameters that determine their shapes such that they coincide in the tangent plane of the surface, then we can, on the other hand, to first order of approximation, reduce the mismatch source to the error. For the affine Gaussian derivative model for spatial receptive fields developed in this article, that matching property is achieved by matching the spatial covariance matrices of the affine Gaussian kernels, in such a way that the support regions of the affine Gaussian kernels may be ellipses in the respective image domains, and may, for example, in the most simple case correspond to circles in the tangent plane of the surface.

Of course, a complementary problems then concerns how to match the actual values of the receptive field parameters in a particular viewing situation. That problem can, however, be seen as a complementary more algorithmic task, in contrast to the fundamental problem of eliminating an otherwise inescapable geometric source of error. In Lindeberg and Gårding (1997) one example of such a computational algorithm was developed for the task of estimating local surface orientation from either monocular or binocular cues. It was demonstrated that it was possible to design an iterative procedure for successively adapting the shapes of the receptive fields, in such a way that it reduced the reconstruction error by about an order of magnitude after a few (2–3) iterations.

While this specific example for illustrating the influence of the backprojected support regions of the receptive fields, when deriving cues to the structure of the world from local image measurements, is developed for the case of spatial affine transformations under a binocular or multi-view viewing situation, a similar geometric problem regarding the backprojected support regions of the receptive fields arises also under monocular projection, as well as for spatio-temporal processing under variations in the relative motion between objects or events in the world and the observer, then concerning the backprojections of the spatio-temporal receptive fields in the 2+1-D video domain to the surfaces of the objects embedded in the 3+1-D world.

A main subject of this paper is to describe a theory for covariant receptive fields under natural image transformations, which makes it possible to perfectly match the backprojected receptive field responses under natural image transformations, approximated by spatial scaling transformations, spatial affine transformations, Galilean transformations and temporal scaling transformations. Since these image transformations may be present in essentially every natural imaging situation, they constitute essential components to include in models of visual perception.

### 1.2. Theory of covariant visual receptive fields

The receptive fields of the neurons in the visual pathway constitute the main computational primitives in the early stages of the visual sensory system. These visual receptive fields integrate and process visual information over local regions over space and time, which is then passed on to higher layers. Capturing the functional properties of visual receptive fields, as well as trying to explain what determines their computational function, constitute key ingredients in understanding vision.

In this work, we will for our theoretical studies build upon the Gaussian derivative model for visual receptive fields, which was initially theoretically proposed by Koenderink (1984), Koenderink and van Doorn (1987, 1992), used for modelling biological receptive fields by Young (1987), Young and Lesperance (2001), Young et al. (2001) and then generalized by Lindeberg (2013, 2021). With regard to the topic of the thematic collection on “Symmetry as a Guiding Principle in Artificial and Brain Neural Networks”, the subject of this article is to describe how symmetry properties in terms of covariance properties under natural image transformations constitute an essential component in that normative theory of visual receptive fields, as well as how such covariance properties may be important with regard to biological vision, specifically to understand the organization of the receptive fields in the primary visual cortex.

It will be shown that a purely spatial version of the studied generalised Gaussian derivative model for visual receptive fields allows for spatial scale covariance and spatial affine covariance, implying that it can, to first order of approximation, handle variations in the distance between objects in the world and the observer, as well as first-order approximations of the geometric transformations induced by viewing the surface of a smooth 3-D object from different viewing distances and viewing directions in the world. It will also be shown that for a more general spatio-temporal version of the resulting generalised Gaussian derivative model, the covariance properties do, in addition, extend to local Galilean transformations, which makes it possible to perfectly handle first-order approximations of the geometric transformations induced by viewing objects in the world under different relative motions between the object and the observer. The spatio-temporal receptive fields in this model do also obey temporal scale covariance, which makes it possible to handle objects that move as well as spatio-temporal events that occur faster or slower in the world. In these ways, the resulting model for visual receptive fields respects the main classes of geometric image transformations in vision.

We argue that if the goal is to build realistic computational models of biological receptive fields, or more generally a computational model of a visual system that should be able to handle general classes of natural image or video data in a robust and stable manner, it is essential that the internal visual representations obey sufficient covariance properties under these classes of geometric image transformations, to, in turn, make it possible to achieve geometric invariance properties at the systems level of the visual system.

We conclude by using predictions from the presented theory to describe implications for biological vision. Specifically, we state experimentally testable hypotheses to explore to what extent the variabilities of receptive field shapes in the primary visual cortex span corresponding variabilities as described by the basic classes of natural image transformations, in terms of spatial scaling transformations, spatial affine transformations, Galilean transformations and temporal scaling transformations. These hypotheses are, in turn, intended for experimentalists to explore and characterize to what extent the primary visual cortex computes a covariant representation of receptive field responses over those classes of natural image transformations.

In this context, we do also describe theoretically how receptive field responses at coarser levels of spatial and temporal scales can be computed from finer scales using cascade smoothing properties over spatial and temporal scales, meaning that it could be sufficient for the visual receptive fields in the first layers to only implement the receptive fields at the finest level of spatial and temporal scales, from which coarser scale representations could then be inferred at higher levels in the visual hierarchy.

## 2. Methods

### 2.1. The generalised Gaussian derivative model for spatial and spatio-temporal receptive fields

In this section, we will describe the generalised Gaussian derivative model for linear receptive fields, in the cases of either (i) image data defined over a purely spatial domain, or (ii) video data defined over a joint spatio-temporal domain, including brief conceptual overviews of how this model can be derived in a principled axiomatic manner, from symmetry properties in relation to the first layers of the visual hierarchy. We do also give pointers to previously established results that demonstrate how these models do qualitatively very well model biological receptive fields in the lateral geniculate nucleus (LGN) and the primary visual cortex (V1), as established by comparisons to results of neurophysiological recordings of visual neurons. This theoretical background will then constitute the theoretical background for the material in Section 3, concerning covariance properties of the visual receptive fields according to the Gaussian derivative model, and the implications of such covariance properties for modelling and explaining the families of receptive field shapes found in biological vision.

#### 2.1.1. Purely spatial models for linear receptive fields

For image data defined over a 2-D spatial domain with image coordinates $x={\left(x_{1},x_{2}\right)}^{T}\in {\mathbb{R}}^{2}$, an axiomatic derivation in Lindeberg (2011); Theorem 5, based on the assumptions of linearity, translational covariance, semi-group^3^ structure over scale and non-creation of new structures from finer to coarser levels of scales in terms of non-enhancement of local extrema^4^, combined with certain regularity assumptions, shows that under evolution of a spatial scale parameter *s*, that reflects the spatial size of the receptive fields, the spatially smoothed image representations *L*(*x*_1_, *x*_2_; *s*) that underlie the output from the receptive fields must satisfy a spatial diffusion equation of the form


(1) $$\begin{array}{l} \partial_{s}L=\frac{1}{2}\nabla_{\left(x_{1},x_{2}\right)}^{T}\left(\Sigma_{\left(x_{1},x_{2}\right)}\nabla_{\left(x_{1},x_{2}\right)}L\right)-\delta_{\left(x_{1},x_{2}\right)}^{T}\nabla_{\left(x_{1},x_{2}\right)}L \end{array}$$

with initial condition *L*(*x*_1_, *x*_2_; 0) = *f*(*x*_1_, *x*_2_), where *f*(*x*_1_, *x*_2_) is the input image, $\nabla_{\left(x_{1},x_{2}\right)}={\left(\partial_{x_{1}},\partial_{x_{2}}\right)}^{T}$ is the spatial gradient vector, Σ_(*x*_1_, *x*_2_)_ is a spatial covariance matrix and δ_(*x*_1_, *x*_2_)_ is a spatial drift vector.

The physical interpretation of this equation is that if we think of the intensity distribution *L* in the image plane as a heat distribution, then this equation describes how the heat distribution will evolve over the virtual time variable *s*, where hot spots will get successively cooler and cold spots will get successively warmer. The evolution will in this sense serve as a spatial smoothing process over the variable *s*, which we henceforth will refer to as a spatial scale parameter of the receptive fields.

The spatial covariance matrix Σ_(*x*_1_, *x*_2_)_ in this equation determines how much spatial smoothing is performed in different directions in the spatial domain, whereas the spatial drift vector δ_(*x*_1_, *x*_2_)_ implies that the smoothed image structures may move spatially over the image domain during the smoothing process. The latter property can, for example, be used for aligning image structures under variations in the disparity field for a binocular visual observer. For the rest of the present treatment, we will, however, focus on monocular viewing conditions and disregard that term in the case of purely spatial image data. For the case of spatio-temporal data, to be considered later, a correspondence to this drift term will on the other hand be used for handling motion, to perform spatial smoothing of objects that change the positions of their projections in the image plane over time, without causing excessive amounts of motion blur, if desirable, as it will be for a receptive field tuned to a particular motion direction.

In terms of the spatial smoothing kernels that underlie the definition of a family of spatial receptive fields, that integrate information over a spatial support region in the image domain, this smoothing process corresponds to smoothing with spatially shifted affine Gaussian kernels of the form


(2) $$\begin{array}{l} g\left(x;\Sigma_{s},\delta_{s}\right)=\frac{1}{2\pi \sqrt{\det\Sigma_{s}}}e^{-{\left(x-\delta_{s}\right)}^{T}\Sigma_{s}^{-1}\left(x-\delta_{s}\right)/2}, \end{array}$$

for a scale dependent spatial covariance matrix Σ_*s*_ and a scale-dependent spatial drift vector δ_*s*_ of the form Σ_*s*_ = *s* Σ_(*x*_1_, *x*_2_)_ and δ_*s*_ = *s* δ_(*x*_1_, *x*_2_)_. Requiring these spatial smoothing kernels to additionally be mirror symmetric around the origin, and removing the parameter *s* from the notation, does, in turn, lead to the regular family of affine Gaussian kernels of the form


(3) $$\begin{array}{l} g\left(x;\Sigma \right)=\frac{1}{2\pi \sqrt{\det\Sigma }}e^{-x^{T}\Sigma^{-1}x/2}. \end{array}$$

Considering that spatial derivatives of the output of affine Gaussian convolution also satisfy the diffusion equation (1), and incorporating scale-normalised derivatives of the form (Lindeberg, 1998)


(4) $$\begin{array}{l} \partial_{\xi^{\alpha }}=s^{\left(\alpha_{1}+\alpha_{2}\right)/m}\partial_{x_{1}}^{\alpha_{1}}\partial_{x_{2}}^{\alpha_{2}}, \end{array}$$

where α = (α_1_, α_2_) denotes the order of differentiation, as well as combining multiple partial derivatives into directional derivative operators according to


(5) $$\begin{array}{l} \partial_{\varphi }^{m}={\left(\cos\alpha \partial_{x_{1}}+\sin\alpha \partial_{x_{2}}\right)}^{m}, \end{array}$$

where we choose the directions φ for computing the directional derivative to be aligned with the directions of the eigenvectors of the spatial covariance matrix Σ, we obtain the following canonical model^5^ for oriented spatial receptive fields of the form [Lindeberg, 2021; Equation (23)]


$$\begin{array}{l} T_{simple}\left(x_{1},x_{2};s,\varphi ,\Sigma ,m\right)=T_{\varphi^{m},norm}\left(x_{1},x_{2};s,\Sigma \right) \end{array}$$

(6) $$\begin{array}{l} =s^{m/2}\partial_{\varphi }^{m}\left(g\left(x_{1},x_{2};s\Sigma \right)\right). \end{array}$$

**Figure 3:**
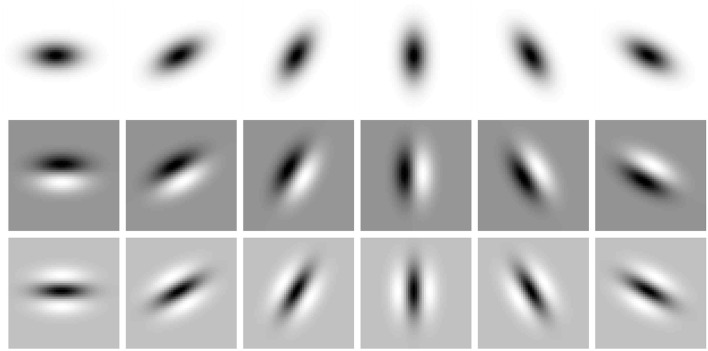
Illustration of the variability of receptive field shapes over spatial rotations and the order of spatial differentiation. This figure shows affine Gaussian kernels *g*(*x*_1_, *x*_2_; Σ) with their directional derivatives ∂_φ_*g*(*x*_1_, *x*_2_; Σ) and ∂_φφ_*g*(*x*_1_, *x*_2_; Σ) up to order two, here with the eigenvalues of Σ being λ_1_ = 64, λ_2_ = 16 and for the image orientations φ = 0, π/6, π/3, π/2, 2π/3, 5π/6. With regard to the classes of image transformations considered in this paper, this figure shows an expansion over the rotation group. In addition, the family of affine Gaussian kernels also comprises an expansion over a variability over the ratio between the eigenvalues of the spatial covariance matrix Σ, that determines the shape of the affine Gaussian kernels, see Figure 4 for complementary illustrations (Horizontal dimension: *x* ∈ [−24, 24]. Vertical dimension: *y* ∈ [−24, 24].).

**Figure 4:**
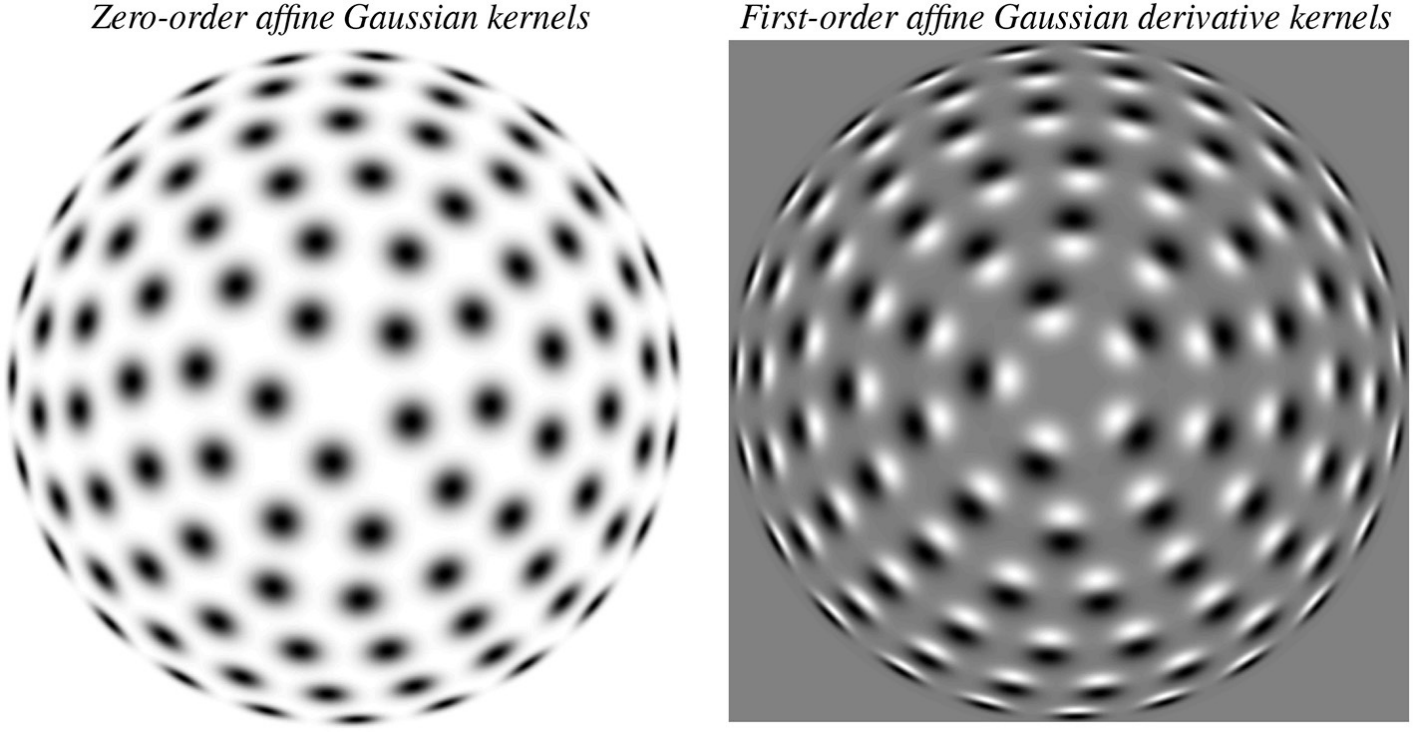
Illustration of the variability of receptive field shapes over spatial affine transformations that also involve a variability of the eccentricity of the receptive fields. (**Left**) Zero-order affine Gaussian kernels for different orientations φ in the image domain as well as for different ratios between the eigenvalues λ_1_ and λ_2_ of the spatial covariance matrix Σ, here illustrated in terms of a uniform distribution of isotropic receptive field shapes on a hemisphere, which will correspond to anisotropic affine Gaussian receptive fields when mapping rotationally symmetric Gaussian receptive fields from the tangent planes of the sphere to the image domain by orthographic projection. (**Right**) First-order directional derivatives of the zero-order affine Gaussian kernels according to that distribution, with the spatial direction for computing the directional derivatives aligned with the orientation of one of the eigenvectors of the spatial covariance matrix Σ that determines the shape of the affine Gaussian kernel. When going from the center of each of these images to the boundaries, the eccentricity, defined as the ratio $\epsilon =\sqrt{\lambda_{min}/\lambda_{max}}$ between the square roots of the smaller and larger eigenvalues λ_*min*_ and λ_*max*_ of Σ varies from 1 to 0, where the receptive fields at the center are maximally isotropic, whereas the receptive fields at the boundaries are maximally anisotropic.

Figure 3 shows examples of such affine Gaussian kernels with their directional derivatives up to order two, for different orientations in the image domain, expressed for one specific choice of the ratio between the eigenvalues of the spatial covariance matrix Σ. More generally, this model also comprises variations of the ratio between the eigenvalues, as illustrated in Figure 4 Left, which visualizes a uniform distribution of zero-order affine Gaussian kernels on a hemisphere. The receptive fields in the latter illustration should then, in turn, be complemented by directional derivatives of such kernels up to a given order of spatial differentiation, see Figure 4 Right for an illustration of such directional derivative receptive fields of order one. In the most idealised version of the theory, one could think of all these receptive fields, with their directional derivatives up to a certain order, as being present at every point in the visual field (if we disregard the linear increase in minimal receptive field size from the fovea towards the periphery in a foveal vision system). With respect to a specific implementation of this model in a specific vision system, it then constitutes a complementary design choice, to sample the variability of that parameter space in an efficient manner.

For rotationally symmetric receptive fields over the spatial domain, a corresponding model can instead be expressed of the form [Lindeberg, 2021; Equation (39)]


(7) $$\begin{array}{l} T_{LGN}\left(x_{1},x_{2}s\right)=\pm s\left(\partial_{x_{1}x_{1}}+\partial_{x_{2}x_{2}}\right)g\left(x_{1},x_{2};s\right). \end{array}$$

In Lindeberg (2013) it is proposed that the purely spatial component of simple cells in the primary visual cortex can be modelled by directional derivatives of affine Gaussian kernels of the form (6); see Figures 16 and 17 in Lindeberg (2021) for illustrations. It is also proposed that the purely spatial component of LGN neurons can be modelled by Laplacians of Gaussians of the form (7); see Figure 13 in Lindeberg (2021).

The affine Gaussian derivative model of simple cells in (6) goes beyond the previous theoretical studies by Koenderink and van Doorn (1987, 1992) and as well as the previous biological modelling work by Young (1987), in that the underlying spatial smoothing kernels in our model are anisotropic, as opposed to isotropic in Young's and Koenderink and van Doorn's models. This anisotropy leads to better approximation of the biological receptive fields, which are more elongated (anisotropic) over the spatial image domain than can be accurately captured based on derivatives of rotationally symmetric Gaussian kernels. The generalised Gaussian derivative model based on affine Gaussian kernels does also enable affine covariance, as opposed to mere scale and rotational covariance for the regular Gaussian derivative model, based on derivatives of the rotationally symmetric Gaussian kernel.

#### 2.1.2. Joint spatio-temporal models for linear receptive fields

For video data defined over a 2+1-D non-causal spatio-temporal domain with coordinates $p={\left(x_{1},x_{2},t\right)}^{T}\in {\mathbb{R}}^{3}$, a corresponding axiomatic derivation, also based on Theorem 5 in Lindeberg (2011), while now formulated over a joint spatio-temporal domain, shows that under evolution over a joint spatio-temporal scale parameter *u*, the spatio-temporally smoothed video representations *L*(*x*_1_, *x*_2_, *t*; *s*, τ, *v*, Σ), that underlie the output from the receptive fields, must satisfy a spatio-temporal diffusion equation of the form


(8) $$\begin{array}{l} \partial_{u}L=\frac{1}{2}\nabla_{\left(x_{1},x_{2},t\right)}^{T}\left(\Sigma_{\left(x_{1},x_{2},t\right)}\nabla_{\left(x_{1},x_{2},t\right)}L\right)-\delta_{\left(x_{1},x_{2},t\right)}^{T}\nabla_{\left(x_{1},x_{2},t\right)}L \end{array}$$

for *u* being some convex combination of the spatial scale parameter *s* and the temporal scale parameter τ, and with initial condition *L*(*x*_1_, *x*_2_, *t*; 0, 0, *v*, Σ) = *f*(*x*_1_, *x*_2_, *t*), where *f*(*x*_1_, *x*_2_, *t*) is the input video, $\nabla_{\left(x_{1},x_{2},t\right)}={\left(\partial_{x_{1}},\partial_{x_{2}},\partial_{t}\right)}^{T}$ is the spatio-temporal gradient, Σ_(*x*_1_, *x*_2_, *t*)_ is a spatio-temporal covariance matrix and δ_(*x*_1_, *x*_2_, *t*)_ is a spatio-temporal drift vector.

If we think of the intensity distribution *L* over joint space-time as a heat distribution, then this equation describes how the heat distribution will evolve over an additional virtual time variable *u* (operating at a higher meta level than the physical time variable *t*), with the spatio-temporal covariance matrix Σ_(*x*_1_, *x*_2_, *t*)_ describing how much smoothing of the virtual heat distribution will be performed in different directions in joint (real) space-time, whereas the spatio-temporal drift vector δ_(*x*_1_, *x*_2_, *t*)_ describes how the image structures may move in joint (real) space-time as function of the virtual variable *u*, which is important for handling the perspective projections of objects that move in the real world.

In Lindeberg (2021); Appendix B.1 it is shown that the solution of this equation can be expressed as the convolution with spatio-temporal kernels of the form


(9) $$\begin{array}{l} T\left(x_{1},x_{2},t;s,\tau ,v,\Sigma \right)=g\left(x_{1}-v_{1}t,x_{2}-v_{2}t;s\Sigma \right)h\left(t;\tau \right), \end{array}$$

where Σ is a spatial covariance matrix, *v* = (*v*_1_, *v*_2_) is a velocity vector and *h*(*t*; τ) is a 1-D temporal Gaussian kernel. Combining this model with spatial directional derivatives in a similar way as for the spatial model, and introducing scale-normalised temporal derivatives (Lindeberg, 2016) of the form


(10) $$\begin{array}{l} \partial_{\zeta }^{n}=\tau^{n/2}\partial_{t}^{n} \end{array}$$

as well as corresponding velocity-adapted temporal derivatives according to


(11) $$\begin{array}{l} \partial_{\bar{t}}=v_{1}\partial_{x_{1}}+v_{2}\partial_{x_{2}}+\partial_{t}, \end{array}$$

then leads to a non-causal spatio-temporal receptive field model of the form [Lindeberg, 2021; Equation (32)]


$$\begin{array}{l} T_{\varphi^{m},{\bar{t}}^{n},norm}\left(x_{1},x_{2},t;s,\tau ,v,\Sigma \right) \end{array}$$

(12) $$\begin{array}{l} =\partial_{\varphi }^{m}\partial_{\bar{t}}^{n}\left(g\left(x_{1}-v_{1}t,x_{2}-v_{2}t;s\Sigma \right)h\left(t;\tau \right)\right). \end{array}$$

Regarding the more realistic case of video data defined over a 2+1-D time-causal spatio-temporal domain, in which the future cannot be accessed, theoretical arguments in Lindeberg (2016, 2021); [Appendix B.2; see Equation (33)] lead to spatio-temporal smoothing kernels of a similar form as in (12),


$$\begin{array}{l} \begin{array}{c} T_{simple}\left(x_{1},x_{2},t;s,\tau ,\varphi ,v,\Sigma ,m,n\right)=T_{\varphi^{m},{\bar{t}}^{n},norm}\left(x_{1},x_{2},t;s,\tau ,v,\Sigma \right) \end{array} \end{array}$$

(13) $$\begin{array}{l} \begin{array}{c} =s_{\varphi }^{m/2}\tau^{n/2}\partial_{\varphi }^{m}\partial_{\bar{t}}^{n}\left(g\left(x_{1}-v_{1}t,x_{2}-v_{2}t;s\Sigma \right)\psi \left(t;\tau ,c\right)\right), \end{array} \end{array}$$

but now with the non-causal temporal Gaussian kernel replaced by a time-causal temporal kernel referred to as the time-causal limit kernel


(14) $$\begin{array}{l} h\left(t;\tau \right)=\psi \left(t;\tau ,c\right), \end{array}$$

defined by having a Fourier transform of the form


(15) $$\begin{array}{l} \hat{\text{Ψ}}\left(\omega ;\tau ,c\right)=\overset{\infty }{\underset{k=1}{\prod }}\frac{1}{1+ic^{-k}\sqrt{c^{2}-1}\sqrt{\tau }\omega }, \end{array}$$

and corresponding the an infinite set of truncated exponential kernels coupled in cascade, with specifically chosen time constants to obtain temporal scale covariance (Lindeberg, 2016, Section 5; Lindeberg, 2023b, Section 3.1).

**Figure 5:**
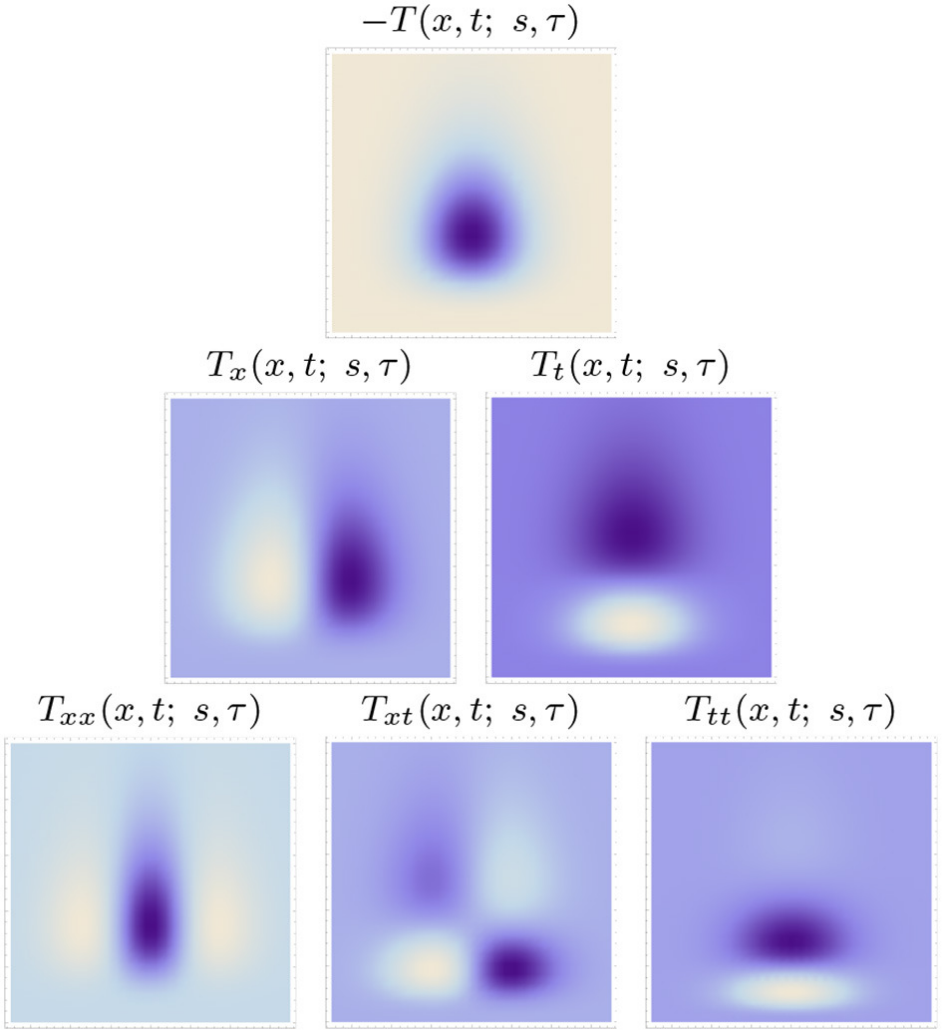
Space-time separable kernels according to the time-causal spatio-temporal receptive field model in (13) up to order two over a 1+1-D spatio-temporal domain for image velocity *v* = 0, using the time-causal limit kernel (14) with distribution parameter $c=\sqrt{2}$ for smoothing over the temporal domain, at spatial scale *s* = 1 and temporal scale τ = 1. (Horizontal dimension: space *x* ∈ [−3.5, 3.5]. Vertical dimension: time *t* ∈ [0, 5].)

**Figure 6:**
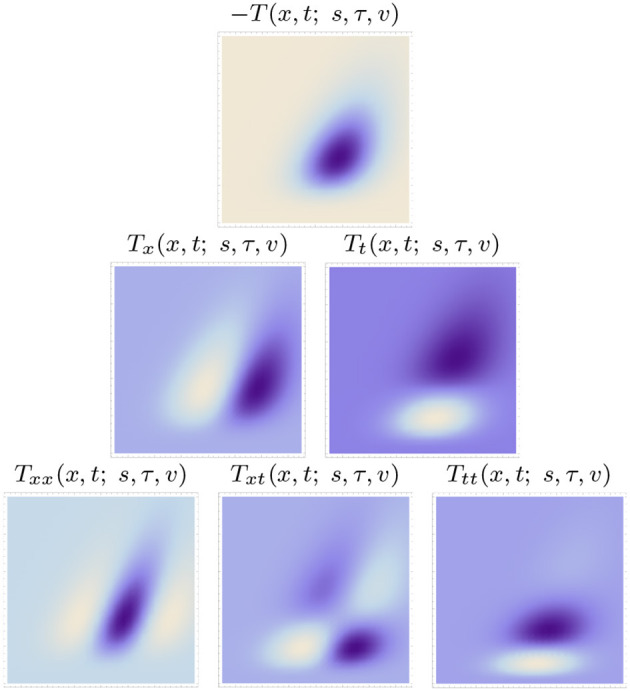
Non-separable spatio-temporal kernels according to the time-causal spatio-temporal receptive field model in (13) up to order two over a 1+1-D spatio-temporal domain for image velocity *v* = 1/2, using the time-causal limit kernel (14) with distribution parameter $c=\sqrt{2}$ for smoothing over the temporal domain, at spatial scale *s* = 1 and temporal scale τ = 1 (Horizontal dimension: space *x* ∈ [−3.5, 3.5]. Vertical dimension: time *t* ∈ [0, 5].).

**Figure 7:**
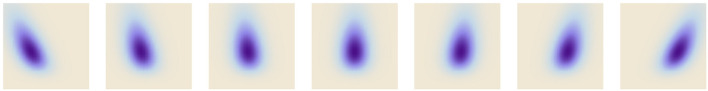
Illustration of the variability of receptive field shapes over Galilean motions. This figure shows zero-order spatio-temporal receptive fields of the form (13), for a self-similar distribution of the velocity values *v*, using Gaussian smoothing over the spatial domain and smoothing with the time-causal limit kernel (14) with distribution parameter $c=\sqrt{2}$ over the temporal domain, in the case of a 1+1-D time-causal spatio-temporal domain, for spatial scale parameter *s* = 1 and temporal scale parameter τ = 4. These primitive spatio-temporal smoothing operations should, in turn, be complemented by spatial differentiation $\partial_{x}^{m}$ and velocity-adapted temporal differentiation operators according to $\partial_{\bar{t}}^{n}={\left(v\partial_{x}+\partial_{t}\right)}^{n}$ for different orders *m* and *n* of spatial and temporal differentiation, respectively, to generate the corresponding family of spatio-temporal receptive fields of the form (13). The central receptive field in this illustration, for zero image velocity is space-time separable, whereas the other receptive fields, for velocity values *v* = −0.4, −0.2, −0.1, 0.1, 0.2 and 0.4 from left to right, are not separable (Horizontal dimension: space *x* ∈ [−4, 4]. Vertical dimension: time *t* ∈ [0, 8].).

Figures 5, 6 show examples of such spatio-temporal receptive fields up to order two, for the case of a 1+1-D spatio-temporal domain. In Figure 5, where the image velocity *v* is zero, the receptive fields are space-time separable, whereas in Figure 6, where the image velocity is non-zero, the receptive fields are not separable over space-time. In the most idealised version of the theory, we could think of these velocity-adapted receptive fields, for all velocity values within some range, as being present at every point in the image domain, see Figure 7 for an illustration.

In this way, the receptive field family will be Galilean covariant, which makes it possible for the vision system to handle observations of moving objects and events in the world, irrespective of the relative motion between the object or the event in the world and the viewing direction.

For rotationally symmetric receptive fields over the spatial domain, a corresponding model can instead be expressed of the form [Lindeberg, 2021; Equation (39)]


$$\begin{array}{l} T_{LGN}\left(x_{1},x_{2},t;s,\tau \right) \end{array}$$

(16) $$\begin{array}{l} =\pm s\tau^{n/2}\left(\partial_{x_{1}x_{1}}+\partial_{x_{2}x_{2}}\right)g\left(x_{1},x_{2};s\right)\partial_{t^{n}}\psi \left(t;\tau ,c\right). \end{array}$$

In Lindeberg (2016) it is proposed that simple cells in the primary visual cortex can be modelled by spatio-temporal kernels of the form (13) for *h*(*t*; τ) = ψ(*t*; *t*) according to (15); see Figure 18 in Lindeberg (2021) for illustrations. It is also proposed that lagged and non-lagged LGN neurons can be modelled by temporal derivatives of Laplacians of Gaussians of the form (16); see Figure 12 in Lindeberg (2021).

These models go beyond the previous modelling work by Young and Lesperance (2001), Young et al. (2001) in that our models are truly time-causal, as opposed to Young's non-causal model, and also that the parameterization of the filter shapes in our model is different, more in line with the geometry of the spatio-temporal image transformations.

#### 2.1.3. Spatio-chromatic and spatio-chrom-temporal receptive field models

Both of the above spatial and spatio-temporal models can be extended from operating on pure grey-level image information to operating on colour image data, by instead applying these models to each channel of a colour-opponent colour space; see Lindeberg (2013, 2021) for further details as well as modelling results. Since the geometric image transformations have the same effect on colour-opponent channels as on grey-level information, we do here in this article, without loss of generality, develop the theory for the case of grey-level images only, while noting that similar algebraic transformation properties will hold for all the channels in a colour-opponent representation of the image or video data.

## 3. Results

### 3.1. Covariance properties under geometric image transformations

In this section, we will describe covariance properties of the above described generalised Gaussian derivative model, in the cases of either (i) spatial receptive fields over a purely spatial domain, or (ii) spatio-temporal receptive fields over a joint spatio-temporal domain.

An initial treatment of this topic, in less developed form, was given in the supplementary material of Lindeberg (2021), however, on a format that may not be easy to digest for a reader with neuroscientific background. In the present treatment, we develop the covariance properties of the spatial and spatio-temporal receptive field models in an extended as well as more explicit manner, that should be more easy to access. Specifically, we incorporate the transformation properties of the full derivative-based receptive field models as opposed to only the transformation properties of the spatial or spatio-temporal smoothing processes in the previous treatment, and also comprising more developed interpretations of these covariance properties with regard to the associated variabilities in image and video data under natural image transformations. Then, in Section 3.2. we will use these theoretical results to formulate predictions with regard to implications for biological vision, including the formulation of a set of testable biological hypotheses as well as needs for characterising the distributions of biological receptive field shapes.

It will be shown that the purely spatial model for simple cells in (16) is covariant under *affine image transformations* of the form


(17) $$\begin{array}{l} x_{R}=\left(\begin{array}{c} x_{R,1}\\ x_{R,2} \end{array}\right)=\left(\begin{array}{cc} a_{11} & a_{12}\\ a_{21} & a_{22} \end{array}\right)\left(\begin{array}{c} x_{L,1}\\ x_{L,2} \end{array}\right)=Ax_{L}, \end{array}$$

which includes the special cases of covariance under *spatial scaling transformations* with


(18) $$\begin{array}{l} A=S_{x}I=\left(\begin{array}{cc} S_{x} & 0\\ 0 & S_{x} \end{array}\right) \end{array}$$

and *spatial rotations* with


(19) $$\begin{array}{l} A=R=\left(\begin{array}{cc} \cos\theta & -\sin\theta \\ \sin\theta & \cos\theta \end{array}\right). \end{array}$$

For the spatial model of LGN neurons (7), affine covariance cannot be achieved, because the underlying spatial smoothing kernels are rotationally symmetric. That model does instead obey covariance under spatial scaling transformations and spatial rotations.

It will also be shown that the joint spatio-temporal model of simple cells in (13) is, in addition to affine covariant, also covariant under *Galilean transformations* of the form


(20) $$\begin{array}{l} p^{\prime }=\left(\begin{array}{c} x_{1}^{\prime }\\ x_{2}^{\prime }\\ t^{\prime } \end{array}\right)=\left(\begin{array}{ccc} 1 & 0 & u_{1}\\ 0 & 1 & u_{2}\\ 0 & 0 & 1 \end{array}\right)\left(\begin{array}{c} x_{1}\\ x_{2}\\ t \end{array}\right)=\left(\begin{array}{c} x_{1}+u_{1}t\\ x_{2}+u_{2}t\\ t \end{array}\right)=Gp \end{array}$$

as well as covariant under *temporal scaling transformations*


(21) $$\begin{array}{l} t^{\prime }=S_{t}t. \end{array}$$

For the joint spatio-temporal model of LGN neurons (16), the covariance properties are, however, restricted to spatial scaling transformations, spatial rotations and temporal scaling transformations.^6^

The real-world significance of these covariance properties is that:

- If we linearize the perspective mapping from a local surface patch on a smooth surface in the world to the image plane, then the deformation of a pattern on the surface to the image plane can, to first order of approximation, be modelled as a local affine transformation of the form (17).
- If we linearize the projective mapping between two views of a surface patch from different viewing directions, then the projections of a pattern on the surface to the two images can, to first order of approximation, be related by an affine transformation of the form (17).
- If we view an object or a spatio-temporal event in the world in two situations, that correspond to different relative velocities in relation to the viewing direction, then the two spatio-temporal image patterns can, to first order of approximation, be related by local Galilean transformations of the form (20).
- If an object moves or an event in the world occurs faster or slower, then the spatio-temporal image patterns arising from that moving object or event are related by a temporal scaling transformation of the form (21).

By the family of receptive fields being covariant under these image transformation implies that the vision system will have the potential ability to perfectly match the output from the receptive field families, given two or more views of the same object or event in the world. In this way, the vision system will have the potential of substantially reducing the measurement errors, when inferring cues about properties in the world from image measurements in terms of receptive fields. For example, in an early work on affine covariant and affine invariant receptive field representations based on affine Gaussian kernels, it was demonstrated that it is possible to design algorithms that reduce the error in estimating local surface orientation from monocular or binocular cues by an order of magnitude, compared to using receptive fields based on the rotationally symmetric Gaussian kernel (Lindeberg and Gårding, 1997; see the experimental results in Tables 1–3).

Based on this theoretical reasoning, in combination with previous experimental support, we argue that it is essential for an artificial or biological vision system to obey sufficient covariance properties, or sufficient approximations thereof, in order to robustly handle the variabilities in image and video data caused by the natural image transformations that arise when viewing objects and events in a complex natural environment.

#### 3.1.1. Formalism to be used

When applying an image transformation between two image domains, not only the spatial or the spatio-temporal representations of the receptive field output need to be transformed, but also the parameters for the receptive field models need to be appropriately matched. In other words, it is necessary to also derive the transformation properties of the spatial scale parameter *s* and the spatial covariance matrix Σ for the spatial model, and additionally the transformation properties of the temporal scale parameter τ and the image velocity *v* for the spatio-temporal model.

In the following subsections, we will derive the covariance properties of the receptive field models under the respective classes of image transformations. To highlight how the parameters are also affected by the image transformations, we will therefore express these covariance properties in terms of explicit image transformations regarding the output from the receptive field models, complemented by explicit commutative diagrams. To reduce the complexity of the expressions, we will focus on the transformation properties of the output from the underlying spatial and spatio-temporal smoothing kernels only.

The output from the actual derivative-based receptive fields can then be obtained by also complementing the transformation properties of the output from the spatial and spatio-temporal smoothing operations with the transformation properties of the respective spatial and temporal derivative operators, where spatial derivatives transform according to


(22) $$\begin{array}{l} \left(\nabla_{\left(x_{L,1},x_{L,2}\right)}L_{L}\right)\left(x_{L}\right)=A^{T}\left(\nabla_{\left(x_{R,1},x_{R,2}\right)}L_{R}\right)\left(x_{R}\right) \end{array}$$

under an affine transformation (17) of the form


(23) $$\begin{array}{l} L_{L}\left(x_{L}\right)=L_{R}\left(x_{R}\right)\;\text{for }x_{R}=Ax_{L}, \end{array}$$

whereas spatio-temporal derivatives transform according to


(24) $$\begin{array}{l} \left(\nabla_{\left(x_{1},y_{1},t\right)}L\right)\left(p\right)=G^{T}\left(\nabla_{\left(x_{1}^{\prime },x_{2}^{\prime },t^{\prime }\right)}L^{\prime }\right)\left(p^{\prime }\right) \end{array}$$

under a Galilean transformation (20) of the form


(25) $$\begin{array}{l} L\left(p\right)=L^{\prime }\left(p^{\prime }\right)\;\text{for }p^{\prime }=Gp \end{array}$$

with $p={\left(x_{1},x_{2},t\right)}^{T}$ and $p^{\prime }={\left(x_{1}^{\prime },x_{2}^{\prime },t^{\prime }\right)}^{T}$.

**Figure 8:**
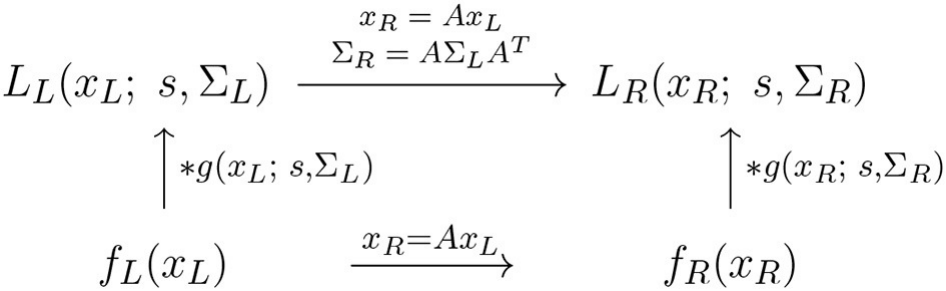
Commutative diagram for the spatial smoothing component in the purely spatial receptive field model (16) under *spatial affine transformations* of the image domain. This commutative diagram, which should be read from the lower left corner to the upper right corner, means that irrespective of the input image *f*_*L*_(*x*_*L*_) is first subject to a spatial affine transformation *x*_*R*_ = *Ax*_*L*_ and then smoothed with an affine Gaussian kernel *g*(*x*_*R*_; *s*, Σ_*R*_), or instead directly convolved with an affine Gaussian kernel *g*(*x*_*L*_; *s*, Σ_*L*_) and then subject to a similar affine transformation, we do then get the same result, provided that the spatial covariance matrices are related according to $\Sigma_{R}=A\Sigma A^{T}$ (and assuming that the spatial scale parameters for the two domain *s*_*R*_ = *s*_*L*_ = *s* are the same).

**Figure 9:**
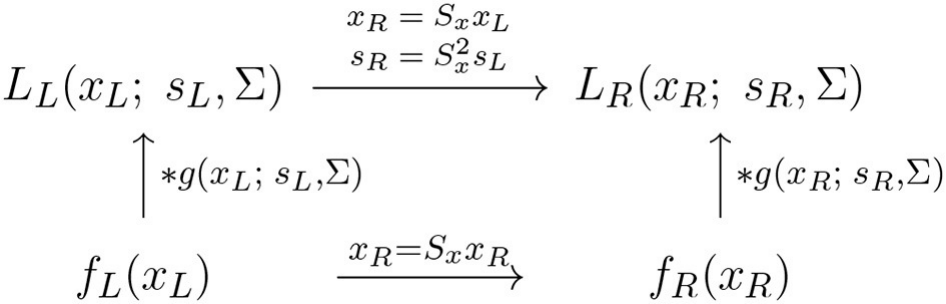
Commutative diagram for the spatial smoothing component in the purely spatial receptive field model (16) under *spatial scaling transformations* of the image domain. This commutative diagram, which should be read from the lower left corner to the upper right corner, means that irrespective of the input image *f*_*L*_(*x*_*L*_) is first subject to spatial scaling transformation *x*_*R*_ = *S*_*x*_*x*_*L*_ and then smoothed with an affine Gaussian kernel *g*(*x*_*R*_; *s*_*R*_, Σ), or instead directly convolved with an affine Gaussian kernel *g*(*x*_*L*_; *s*_*L*_, Σ) and then subject to a similar affine transformation, we do then get the same result, provided that the spatial scale parameters are related according to $s_{R}=S_{x}^{2}s_{L}$ (and assuming that the spatial covariance matrices for the two image domains are the same Σ_*R*_ = Σ_*L*_ = Σ).

**Figure 10:**
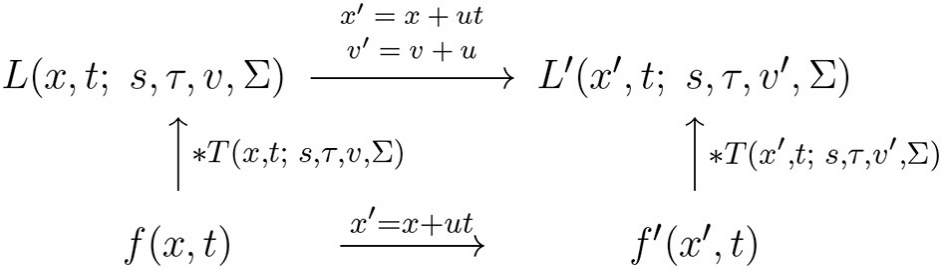
Commutative diagram for the spatio-temporal smoothing component in the joint spatio-temporal receptive field model (13) under *Galilean transformations* over a spatio-temporal video domain. This commutative diagram, which should be read from the lower left corner to the upper right corner, means that irrespective of the input video *f*(*x, t*) is first subject to Galilean transformation *x*′ = *x*′+*u* and then smoothed with a spatio-temporal kernel kernel *T*(*x*′, *t*; *s*, τ, *v*′, Σ), or instead directly convolved with the spatio-temporal smoothing kernel *T*(*x, t*; *s*, τ, *v*, Σ) and then subject to a similar Galilean transformation, we do then get the same result, provided that the velocity parameters of the spatio-temporal smoothing kernels are related according to *v*′ = *v* + *u* (and assuming that the other parameters in the spatio-temporal receptive field models are the same).

**Figure 11:**
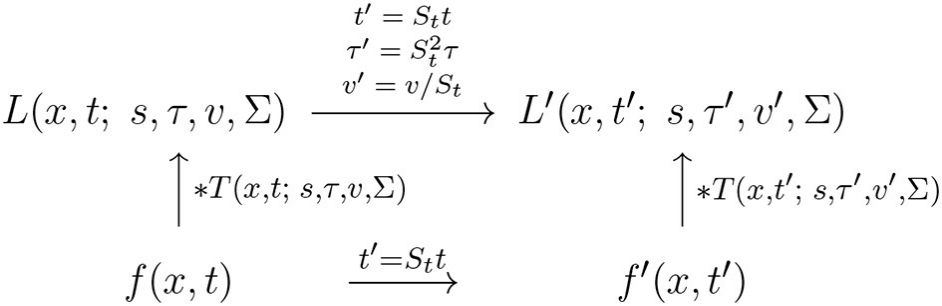
Commutative diagram for the spatio-temporal smoothing component in the joint spatio-temporal receptive field model (13) under *temporal scaling transformations* over a spatio-temporal video domain. This commutative diagram, which should be read from the lower left corner to the upper right corner, means that irrespective of the input video *f*(*x, t*) is first subject to a temporal scaling transformation $t^{\prime }=S_{t}t$ and then smoothed with a spatio-temporal kernel kernel *T*(*x, t*′; *s*, τ′, *v*′, Σ), or instead directly convolved with the spatio-temporal smoothing kernel *T*(*x, t*; *s*, τ, *v*, Σ) and then subject to a similar temporal scaling transformation, we do then get the same result, provided that the temporal scale parameters as well as the velocity parameters of the spatio-temporal smoothing kernels are matched according to $\tau^{\prime }=S_{t}^{2}t$ and $v^{\prime }=v/S_{t}$ (and assuming that the other parameters in the spatio-temporal receptive field models are the same).

**Figure 12:**
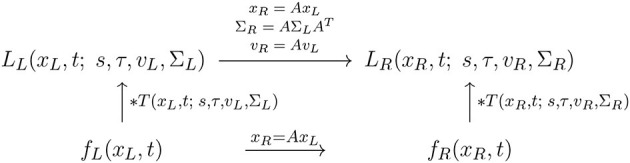
Commutative diagram for the spatio-temporal smoothing component in the joint spatio-temporal receptive field model (13) under *spatial affine transformations* over a spatio-temporal video domain. This commutative diagram, which should be read from the lower left corner to the upper right corner, means that irrespective of the input video *f*(*x*_*L*_, *t*) is first subject to a spatial affine transformation *x*_*R*_ = *Ax*_*L*_ and then smoothed with a spatio-temporal kernel kernel *T*(*x*_*R*_, *t*; *s*, τ, *v*_*R*_, Σ_*R*_), or instead directly convolved with the spatio-temporal smoothing kernel *T*(*x*_*L*_, *t*; *s*, τ, *v*_*L*_, Σ_*L*_) and then subject to a similar temporal scaling transformation, we do then get the same result, provided that the spatial covariance matrices as well as the velocity parameters of the spatio-temporal smoothing kernels are matched according to $\Sigma_{R}=A\Sigma_{L}A^{T}$ and *v*_*R*_ = *Av*_*L*_ (and assuming that the other parameters in the spatio-temporal receptive field models are the same).

**Figure 13:**
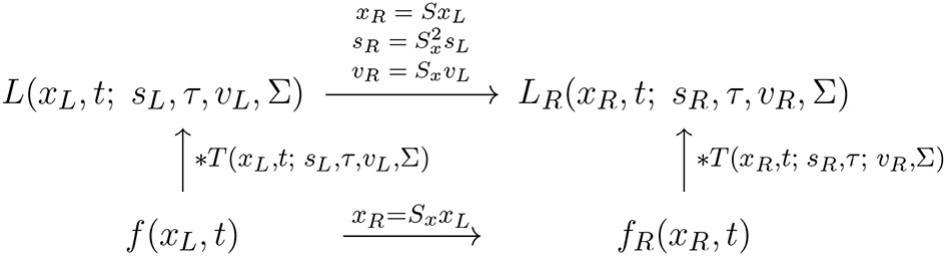
Commutative diagram for the spatio-temporal smoothing component in the joint spatio-temporal receptive field model (13) under *spatial scaling transformations* over a spatio-temporal video domain. This commutative diagram, which should be read from the lower left corner to the upper right corner, means that irrespective of the input video *f*(*x*_*L*_, *t*) is first subject to a spatial scaling transformation *x*_*R*_ = *S*_*x*_*x*_*L*_ and then smoothed with a spatio-temporal kernel kernel *T*(*x*_*R*_, *t*; *s*_*R*_, τ, *v*_*R*_, Σ), or instead directly convolved with the spatio-temporal smoothing kernel *T*(*x*_*L*_, *t*; *s*_*L*_, τ, *v*_*L*_, Σ) and then subject to a similar temporal scaling transformation, we do then get the same result, provided that the spatial scale parameters as well as the velocity parameters of the spatio-temporal smoothing kernels are matched according to $s_{R}=S_{x}^{2}s_{L}$ and *v*_*R*_ = *S*_*x*_*v*_*L*_ (and assuming that the other parameters in the spatio-temporal receptive field models are the same).

By necessity, the treatment that follows next in this section will be somewhat technical. The hasty reader may skip the mathematical derivations in the following text and instead focus on the resulting commutative diagrams in Figures 8–13, which describe the essence of the transformation properties of the receptive fields in the generalised Gaussian derivative model.

#### 3.1.2. Covariance properties for the purely spatial receptive fields

##### 3.1.2.1. Transformation property under spatial affine transformations

To model the essential effect of the receptive field output in terms of the spatial smoothing transformations applied to two spatial image patterns *f*_*L*_(*x*_*L*_) and *f*_*R*_(*x*_*R*_), that are related according to a spatial affine transformation (17) of the form


(26) $$\begin{array}{l} x_{R}=Ax_{L}, \end{array}$$

let us define the corresponding spatially smoothed representations *L*_*L*_(*x*_*L*_; *s*, Σ_*L*_) and *L*_*R*_(*x*_*L*_; *s*, Σ_*R*_) obtained by convolving *f*_*L*_(*x*_*L*_) and *f*_*R*_(*x*_*R*_) with affine Gaussian kernels of the form (3), using spatial scale parameters *s*_*L*_ and *s*_*R*_ as well as spatial covariance matrices Σ_*L*_ and Σ_*R*_, respectively.

Then, according to Lindeberg and Gårding (1997); Section 4.1, these spatially smoothed representations^7^ are related according to


(27) $$\begin{array}{l} L_{L}\left(x_{L};s,\Sigma_{L}\right)=L_{R}\left(x_{R};s,\Sigma_{R}\right) \end{array}$$

for


(28) $$\begin{array}{l} \Sigma_{R}=A\Sigma_{L}A^{T}, \end{array}$$

which constitutes an affine covariant transformation property, as illustrated in the commutative diagram in Figure 8. Specifically, the affine covariance property implies that for every receptive field output from the image *f*_*L*_(*x*_*L*_) computed with spatial covariance matrix Σ_*L*_, there exists a corresponding spatial covariance matrix Σ_*R*_, such that the receptive field output from the image *f*_*R*_(*x*_*R*_) using that covariance matrix can be perfectly matched to the receptive field output from image *f*_*L*_(*x*_*L*_) using the spatial covariance matrix Σ_*L*_.

This property thus means that the family of spatial receptive fields will, up to first order of approximation, have the ability to handle the image deformations induced between the perspective projections of multiple views of a smooth local surface patch in the world.

For the spatial LGN model in (7), a weaker rotational covariance property holds, implying that under a spatial rotation


(29) $$\begin{array}{l} x_{R}=Rx_{L}, \end{array}$$

where *R* is a 2-D rotation matrix, the corresponding spatially smoothed representations are related according to


(30) $$\begin{array}{l} L_{L}\left(x_{L};s\right)=L_{R}\left(x_{R};s\right). \end{array}$$

This property implies that image structures arising from projections of objects that rotate around an axis aligned with the viewing direction are handled in a structurally similar manner.

##### 3.1.2.2. Transformation property under spatial scaling transformations

In the special case when the spatial affine transformation represented by the affine matrix *A* reduces to a scaling transformation, having scaling matrix *S* with scaling factor *S*_*x*_ according to (18)


(31) $$\begin{array}{l} x_{R}=S_{x}x_{L}, \end{array}$$

the above affine covariance relation reduces to the form


(32) $$\begin{array}{l} L_{R}\left(x_{R};s_{R},\Sigma \right)=L_{L}\left(x_{L};s_{L},\Sigma \right), \end{array}$$

provided that the spatial scale parameters are related according to


(33) $$\begin{array}{l} s_{R}=S_{x}^{2}s_{L} \end{array}$$

and assuming that the spatial covariance matrices are the same


(34) $$\begin{array}{l} \Sigma_{R}=\Sigma_{L}=\Sigma . \end{array}$$

With regard to the previous treatment in Section 2.1.1, this reflects the scale covariance property of the spatial smoothing transformation underlying the simple cell model in (6), as illustrated in the commutative diagram in Figure 9.

A corresponding spatial scale covariance property does also hold for the spatial smoothing component in the spatial LGN model in (7), and is of the form


(35) $$\begin{array}{l} L_{R}\left(x_{R};s_{R}\right)=L_{L}\left(x_{L};s_{L}\right), \end{array}$$

provided that the spatial scale parameters are related according to


(36) $$\begin{array}{l} s_{R}=S_{x}^{2}s_{L}. \end{array}$$

This scale covariance property implies that the spatial receptive field family will, up to first order of approximation, have the ability to handle the image deformations induced by viewing an object from different distances relative to the observer, as well as handling objects in the world that have similar spatial appearance, while being of different physical size.

#### 3.1.3. Covariance properties for the joint spatio-temporal receptive fields

##### 3.1.3.1. Transformation property under Galilean transformations

To model the essential effect of the receptive field output in terms of the spatio-temporal smoothing transformation applied to two spatio-temporal video patterns *f*(*p*) and *f*′(*p*′), that are related according to a Galilean transformation (20) according to


(37) $$\begin{array}{l} f^{\prime }\left(x^{\prime },t^{\prime }\right)=f\left(x,t\right) \end{array}$$

for *t*′ = *t*′ and


(38) $$\begin{array}{l} x^{\prime }=x+ut, \end{array}$$

let us define the corresponding spatio-temporally smoothed video representations^8^ *L*(*x, t*; *s*, τ, *v*, Σ) and *L*′(*x*′, *t*′; *s*′, τ′, *v*′, Σ′) obtained by convolving *f*(*p*) and *f*′(*p*) with spatio-temporal smoothing kernels of the form (9) using spatial scale parameters *s* and *s*′, temporal scale parameters τ and τ′, velocity vectors *v* and *v*′, and spatial covariance matrices Σ and Σ′, respectively:


(39) $$\begin{array}{l} \begin{array}{c} L\left(x,t;s,\tau ,v,\Sigma \right)=T\left(x,t;s,\tau ,v,\Sigma \right)*f\left(x,t\right), \end{array} \end{array}$$

(40) $$\begin{array}{l} \begin{array}{c} L^{\prime }\left(x^{\prime },t^{\prime };s^{\prime },\tau^{\prime },v^{\prime },\Sigma^{\prime }\right)=T\left(x^{\prime },t^{\prime };s^{\prime },\tau^{\prime },v^{\prime },\Sigma^{\prime }\right)*f\left(x^{\prime },t^{\prime }\right). \end{array} \end{array}$$

By relating these two representations to each other, according to a change of variables, it then follows that the spatio-temporally smoothed video representations are related according to the Galilean covariance property


(41) $$\begin{array}{l} L^{\prime }\left(x^{\prime },t^{\prime };s^{\prime },\tau^{\prime },v^{\prime },\Sigma^{\prime }\right)=L\left(x,t;s,\tau ,v,\Sigma \right), \end{array}$$

provided that the velocity parameters for the two different receptive field models are related according to


(42) $$\begin{array}{l} v^{\prime }=v+u \end{array}$$

and assuming that the other receptive field parameters are the same, *i.e*., that *s*′ = *s*, Σ′ = Σ and τ′ = τ, see the commutative diagram in Figure 10 for an illustration.

The regular LGN model (7), based on space-time separable receptive fields, is not covariant under Galilean transformations. Motivated by the fact that there are neurophysiological results showing that that there also exist neurons in the LGN that are sensitive to motion directions (Ghodrati et al., 2017, Section 1.4.2), we could, however, also formulate a tentative Galilean covariant receptive field model for LGN neurons, having rotationally symmetric receptive fields over the spatial domain, according to


$$\begin{array}{l} h_{LGN}\left(x_{1},x_{2},t;s,\tau ,v\right)= \end{array}$$

(43) $$\begin{array}{l} \pm s\tau^{n/2}\left(\partial_{x_{1}x_{1}}+\partial_{x_{2}x_{2}}\right)\partial_{{\bar{t}}^{n}}\left(g\left(x-v_{1}t,y-v_{2}t;s\right)\psi \left(t;\tau ,c\right)\right). \end{array}$$

Then, the underlying purely spatio-temporally smoothed representations, disregarding the Laplacian operator $\nabla_{x_{1},x_{2})}^{2}=\partial_{x_{1}x_{1}}+\partial_{x_{2}x_{2}}$ and the temporal derivative operator $\partial_{{\bar{t}}^{n}}$, will under a Galilean transformation (38) be related according to


(44) $$\begin{array}{l} L^{\prime }\left(x^{\prime },t^{\prime };s^{\prime },\tau^{\prime },v^{\prime }\right)=L\left(x,t;s,\tau ,v\right), \end{array}$$

provided that the velocity parameters for the two different receptive field models are related according to


(45) $$\begin{array}{l} v^{\prime }=v+u, \end{array}$$

and assuming that the other receptive field parameters are the same, *i.e*., that *s*′ = *s* and τ′ = τ.

These two Galilean covariance properties mean that the corresponding families of spatio-temporal receptive fields will, up to first order of approximation, have the ability to handle the image deformations induced between objects that move, as well as spatio-temporal events that occur, with different relative velocities between the object/event and the observer.

##### 3.1.3.2. Transformation property under temporal scaling transformations

To model the essential effect of the receptive field output in terms of the spatio-temporal smoothing transformations applied to two spatio-temporal video patterns *f*(*p*) and *f*′(*p*′), that are related according to a temporal scaling transformation (21) according to


(46) $$\begin{array}{l} f^{\prime }\left(x^{\prime },t^{\prime }\right)=f\left(x,t\right) \end{array}$$

for *x*′ = *x* and


(47) $$\begin{array}{l} t^{\prime }=S_{t}^{2}t, \end{array}$$

with the temporal scaling factor *S*_*t*_ restricted^9^ to being an integer^10^ power of the distribution parameter *c* > 1 of the time-causal limit kernel ψ(*t*; τ, *c*) in the time-causal spatio-temporal receptive field model


(48) $$\begin{array}{l} S_{t}=c^{i}, \end{array}$$

let us analogously to above define the corresponding spatio-temporally smoothed video representations *L*(*x, t*; *s*, τ, *v*, Σ) and *L*′(*x*′, *t*′; *s*′, τ′, *v*′, Σ′, *v*′) of *f*(*p*) and *f*′(*p*′), respectively, according to (39) and (40).

Then, due to the scale-covariant property of the time-causal limit kernel [Lindeberg, 2016, Equation (47); Lindeberg, 2023b, Equation (34)], these spatio-temporally smoothed video representations are related according to the temporal scale covariance property


(49) $$\begin{array}{l} L^{\prime }\left(x^{\prime },t^{\prime };s^{\prime },\tau^{\prime },v^{\prime },\Sigma^{\prime }\right)=L\left(x,t;s,\tau ,v,\Sigma \right), \end{array}$$

provided that the temporal scale parameters and the velocity parameters for the two receptive field models are matched according to


(50) $$\begin{array}{l} \tau^{\prime }=S_{t}^{2}\tau \;\text{and }v^{\prime }=v/S, \end{array}$$

and assuming that the other receptive field parameters are the same, *i.e*., that *s*′ = *s* and Σ′ = Σ, see the commutative diagram in Figure 11 for an illustration.

A corresponding temporal covariance property does also hold for the space-time separable spatio-temporal LGN model in (16), of the form


(51) $$\begin{array}{l} L^{\prime }\left(x,t^{\prime };s,\tau^{\prime }\right)=L_{L}\left(x,t;s,\tau \right), \end{array}$$

provided that the temporal scale parameters are related according to


(52) $$\begin{array}{l} \tau^{\prime }=S_{t}^{2}\tau . \end{array}$$

These temporal scaling covariance properties mean that the families of spatio-temporal receptive fields will be able to handle objects that move and events that occur faster or slower in the world.

##### 3.1.3.3. Transformation property under spatial affine transformations

To model the essential effect of the receptive field output in terms of the spatio-temporal smoothing transformation applied to two spatio-temporal video patterns *f*(*p*_*L*_) and *f*(*p*_*R*_), that are related according to a spatial affine transformation (17) according to


(53) $$\begin{array}{l} x_{R}=Ax_{L}, \end{array}$$

let us analogously to in Section 3.1.3.1. define the corresponding spatio-temporally smoothed video representations *L*_*L*_(*x*_*L*_, *t*_*L*_; *s*_*L*_, τ_*L*_, *v*_*L*_, Σ_*L*_) and *L*_*R*_(*x*_*R*_, *t*_*R*_; *s*_*R*_, τ_*T*_, *v*_*R*_, Σ_*R*_) of *f*(*p*_*L*_) = *f*(*p*) and $f\left(p_{R}\right)=f^{\prime }\left(p\right)$, respectively, according to (39) and (40).

Then, based on similar transformation properties as are used for deriving the affine covariance property of the purely spatial receptive field model in Section 3.1.2.1., it follows that these spatio-temporally smoothed video representations are related according to the spatial affine covariant transformation property


(54) $$\begin{array}{l} L_{R}\left(x_{R},t_{R};s_{R},\tau_{R},v_{R},\Sigma_{R}\right)=L_{L}\left(x_{L},t_{L};s_{L},\tau_{L},v_{L},\Sigma_{L}\right) \end{array}$$

for


(55) $$\begin{array}{l} \Sigma_{R}=A\Sigma_{L}A^{T}\;\text{and }v_{R}=Av_{L}, \end{array}$$

provided that the other receptive field parameters are the same, *i.e*., that *s*_*R*_ = *s*_*L*_, τ_*R*_ = τ_*L*_ and *v*_*R*_ = *v*_*L*_.

With regard to the previous treatment in Section 2.1.1, this property reflects the spatial affine covariance property of the spatio-temporal smoothing transformation underlying the simple cell model in (13), as illustrated in the commutative diagram in Figure 12.

This affine covariance property implies that, also under non-zero relative motion between an object or event in the world and the observer, the family of spatio-temporal receptive fields will have the ability to, up to first order of approximation, handle the image deformations caused by viewing the surface pattern of a smooth surface in the world under variations in the viewing direction relative to the observer.

For the spatio-temporal LGN model with velocity adaptation in (43), a weaker rotational covariance property holds, implying that under a spatial rotation


(56) $$\begin{array}{l} x_{R}=Rx_{L}, \end{array}$$

where *R* is a 2-D rotation matrix, the corresponding spatially smoothed representations are related according to


(57) $$\begin{array}{l} L_{L}\left(x_{L},t;s,\tau ,v_{L}\right)=L_{R}\left(x_{R},t;s,\tau ,v_{R}\right), \end{array}$$

provided that


(58) $$\begin{array}{l} v_{R}=Rv_{L}. \end{array}$$

For the spatio-temporal LGN model without velocity adaptation in (16), a similar result holds, with the conceptual difference that *v*_*R*_ = *v*_*L*_ = 0.

As for the previously studied purely spatial case, these rotational covariance properties imply that image structures arising from projections of objects that rotate around an axis aligned with the viewing direction are handled in a structurally similar manner.

##### 3.1.3.4. Transformation property under spatial scaling transformations

In the special case when the spatial affine transformation represented the affine matrix *A* reduces to a scaling transformation with a spatial scaling matrix *S*, with spatial scaling factor *S*_*x*_ according to (18)


(59) $$\begin{array}{l} x_{R}=S_{x}x_{L}, \end{array}$$

the above spatial affine covariance relation reduces to the form


(60) $$\begin{array}{l} L_{R}\left(x_{R},t;s_{R},\tau ,v_{R},\Sigma \right)=L_{L}\left(x_{L},t;s_{L},\tau ,v_{L},\Sigma \right), \end{array}$$

provided that the spatial scale parameters and the velocity vectors are related according to


(61) $$\begin{array}{l} x_{R}=S_{x}^{2}x_{L}\;\text{and }v_{R}=S_{x}v, \end{array}$$

and assuming that the other receptive field parameters are the same, *i.e*., that τ_*R*_ = τ_*L*_ = τ, Σ_*R*_ = Σ_*L*_ = Σ and *v*_*R*_ = *v*_*L*_.

With regard to the previous treatment in Section 2.1.1, this instead reflects the scale covariance property of the spatio-temporal smoothing transformation underlying the simple cell model in (6), as illustrated in the commutative diagram in Figure 13.

A corresponding spatial scale covariance property does also hold for the space-time separable spatio-temporal LGN model in (16), of the form


(62) $$\begin{array}{l} L_{R}\left(x_{R},t;s_{R},\tau \right)=L_{L}\left(x_{L},t;s_{L},\tau \right), \end{array}$$

provided that the spatial scale parameters are related according to


(63) $$\begin{array}{l} x_{R}=S_{x}^{2}x_{L}. \end{array}$$

Similar to the previous spatial scale covariance property for purely spatial receptive fields in Section 3.1.2.2, this spatial covariance property means that the spatio-temporal receptive field family will, up to first order of approximation, have the ability to handle the image deformations induced by viewing an object from different distances relative to the observer, as well as handling objects in the world that have similar spatial appearance, while being of different physical size.

### 3.2. Implications of the theory for biological vision

Concerning biological implications of the presented theory, it is sometimes argued that the oriented simple cells in the primary visual cortex serve as mere edge detectors. In view of the presented theory, the oriented receptive fields of the simple cells can, on the other hand, also be viewed as populations of receptive fields, that together make it possible to capture local image deformations in the image domain, to, in turn, serve as a cue for deriving cues to surface orientation and surface shape in the world. In addition, the spatio-temporal dependencies of the simple cells are also essential to handle objects that move as well as spatio-temporal events that occur, with possibly different relative motions in relation to the observer.

According to the presented theory, the spatial and spatio-temporal receptive fields are expanded over their associated filter parameters, whereby the population of receptive fields becomes able to handle the different classes of locally linearised image and video deformations. This is done in such a way that the output from the receptive fields can be perfectly matched under these image and video transformations, provided that the receptive field parameters are properly matched to the actual transformations that occur in a particular imaging situation. Specifically, an interesting follow-up question of this work to biological vision research concerns how well biological vision spans corresponding families of receptive fields, as predicted by the presented theory.

**Figure 14:**
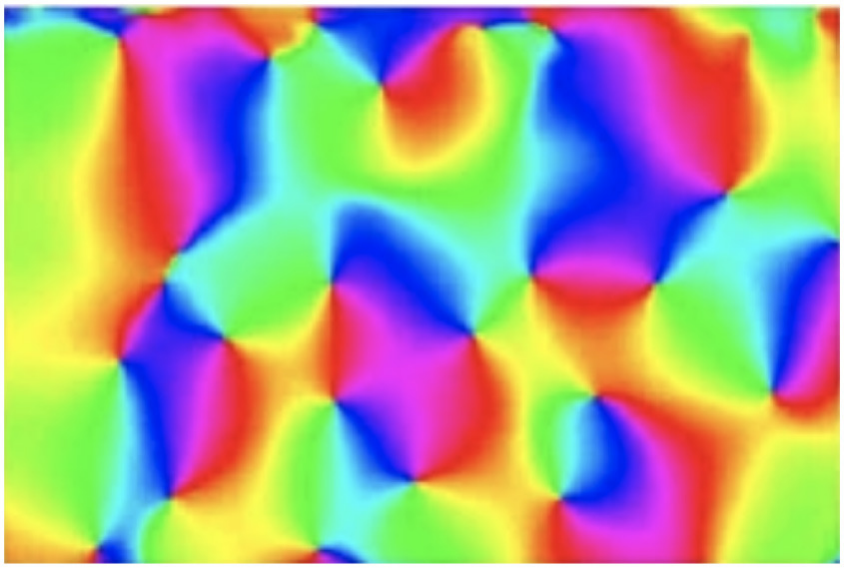
Orientation map in the primary visual cortex (in cat area 17), as recorded by Koch et al. (2016) (OpenAccess), which by a colour coding shows how the preferred spatial orientations for the visual neurons vary spatially over the cortex. In terms of the theory presented in this article, regarding connections between the influence of natural image transformation on image data and the shapes of the visual receptive fields of the neurons that process the visual information, these results can be taken as support that the population of visual receptive fields in the visual cortex perform an expansion over the group of spatial rotations. Let us take into further account that the receptive fields of the neurons near the pinwheels are reported to be comparably isotropic, whereas the receptive fields of the neurons further away from the pinwheels are more anisotropic. Then, we can, from the prediction of an expansion over affine covariance matrices of directional derivatives of affine Gaussian kernels, proposed as a model for the spatial component of simple cells, raise the question if the population of receptive fields can also be regarded as spanning some larger subset of the affine group than mere rotations. If the receptive fields of the neurons span a larger part of the affine group, involving non-uniform scaling transformations that would correspond to spatial receptive fields with different ratios between the characteristic lengths of the receptive fields in the directions of the orientation of the receptive field and its orthogonal direction, then they would have better ability to derive properties, such as the surface orientation and the surface shape of objects in the world, compared to a population of receptive fields that does not perform such an expansion.

In the study of orientation maps of receptive field families around pinwheels (Bonhoeffer and Grinvald, 1991), it has been found that for higher mammals, oriented receptive fields in the primary visual cortex are laid out with a specific organization regarding their directional distribution, in that neurons with preference for a similar orientation over the spatial domain are grouped together, and in such a way that the preferred orientation varies continuously around the singularities in the orientation maps known as pinwheels (see Figure 14). First of all, such an organization shows that biological vision performs an explicit expansion over the group of spatial rotations, which is a subgroup of the affine group.

Beyond variations in mere orientation selectivity of neurons, neurophysiological investigations by Blasdel (1992) have, however, also shown that the degree of orientation selectivity varies regularly over the cortex, and is different near *vs*. further away from the center of a pinwheel; see also Nauhaus et al. (2008) and Koch et al. (2016). Specifically, the orientation selectivity is lowest near the positions of the centers of the pinwheels, and then increases with the distance from the pinwheel. In view of the spatial model for simple cells (6), such a behaviour would be a characteristic property, if the spatial receptive fields would be laid out over the cortex according to a distribution over the spatial covariance matrices of the affine Gaussian kernels, which determine the purely spatial smoothing component in the spatial receptive field model.

For the closest to isotropic affine Gaussian kernels, a small perturbation of the spatial covariance matrix of the spatial smoothing kernel could cause a larger shift in the preferred orientation than for a highly anisotropic Gaussian kernel. The singularity (the pinwheel) in such a model would therefore correspond to the limit case of a rotationally symmetric Gaussian kernel, alternatively an affine Gaussian kernel as near as possible to a rotationally symmetric Gaussian kernel, within some complementary constraint not modelled here, if the parameter variation in the biological system does not reach the same limits as in our idealised theoretical model.

Compare with the orientation maps that would be generated from the distribution of spatial receptive fields shown in Figure 4, although do note that in that illustration, the same spatial orientation is represented at two opposite spatial positions in relation to the origin, whereas the biological orientation maps around the pinwheels only represent a spatial orientation once. That minor technical problem can, however, be easily fixed, by mapping the angular representation in Figure 4 to a double angle representation, which would then identify the directional derivatives of affine Gaussian kernels with opposite polarity, in other words kernels that correspond to flipping the sign of the affine Gaussian derivatives.

An interesting follow-up question for biological vision research does thus concern if it can be neurophysiologically established if the distribution of the spatial shapes of the the spatial smoothing component of the simple cells spans a larger part of the affine group than a mere expansion over spatial rotations?^11^ What would then the distribution be over different eccentricities, *i.e*, different ratios^12^ between the eigenvalues of the spatial covariance matrix Σ, if the spatial component of each simple cells is modelled as a directional derivative (of a suitable order) of an affine Gaussian kernel? Note, in relation to the neurophysiological measurements by Nauhaus et al. (2008), which show a lower orientation selectivity at the pinwheels and increasing directional selectivity when moving further away from the pinwheel, that the spatial receptive fields based on the maximally isotropic affine Gaussian kernels in the centers of Figure 4 (for eccentricity ϵ = 1) would have the lowest degree of orientation selectivity, whereas the spatial receptive fields towards the periphery (for ϵ decreasing towards 0) would have the highest degree of orientation selectivity, see Lindeberg (2023a)^13^ for an in-depth treatment of this topic.

If an expansion over eccentricities for simple cells could be established over the spatial domain, it would additionally be interesting to investigate if such an expansion would be coupled also to the expansion over image velocities *v* in space-time^14^ for joint spatio-temporal receptive fields, or if a potential spatial expansion over eccentricities in the affine group (alternatively over some other subset of the affine group) is decoupled from the image velocities in the Galilean group. If those expansions are coupled, then the dimensionality of the parameter space would be substantially larger, while the dimensionality over two separate expansions over eccentricities *vs*. motion directions would be substantially lower. Is it feasible for the earliest layers of receptive field to efficiently represent those dimensions of the parameter space jointly, which would then enable higher potential accuracy in the derivation of cues to local surface orientation and surface shape from moving objects that are not fixated by the observer, and thus moving with non-zero image velocity relative to the viewing direction. Or do efficiency arguments call for a separation of those dimensions of the parameter space, into separate spatial and motion pathways, so that accurate surface orientation and shape estimation can only be performed with respect to viewing directions that are fixated by the observer in relation to a moving object?

It would additionally be highly interesting to characterize to what extent the early stages in the visual system perform expansions of receptive fields over multiple spatial and temporal scales. In the retina, the spatial receptive fields mainly capture a lowest spatial scale level, which increases linearily with the distance from the fovea (see Lindeberg, 2013, Section 7). Experimental evidence do on the other hand demonstrate that biological vision achieves scale invariance over wide ranges of scale (Biederman and Cooper, 1992; Ito et al., 1995; Logothetis et al., 1995; Furmanski and Engel, 2000; Hung et al., 2005; Isik et al., 2013). Using the semi-group properties of the rotationally symmetric as well as the affine Gaussian kernels,^15^ it is in principle possible to compute coarser scale representations from finer scale levels by adding complementary spatial smoothing stages in cascade. The time-causal limit kernel (14) used for temporal smoothing in our spatio-temporal receptive field model does also obey a cascade smoothing property over temporal scales, which makes it possible to compute representations at coarser temporal scales from representations at finer spatial scales, by complementary (time-causal) temporal filtering, in terms of first-order temporal integrators coupled in cascade^16^ (Lindeberg, 2023b). Do the earliest layers in a biological visual system explicitly represent the image and video data by expansions of receptive fields over multiple spatial and temporal scales, or do the earliest stages in the vision system instead only represent a lowest range of spatial and temporal scales explicitly, to then handle coarser spatial and temporal scales by other mechanisms?

The generalised Gaussian derivative model for visual receptive fields can finally be seen as biologically plausible, in the respect that the computations needed to perform the underlying spatial smoothing operations and spatial derivative computations can be performed by local spatial computations. The spatial smoothing is modelled by diffusion equations (1), which can be implemented by local computations based on connections between neighbours, and thus be performed by groups of neurons that interact with each other spatially by local connections. Spatial derivatives can also be approximated by local nearest neighbour computations. The temporal smoothing operation in the time-causal model based on smoothing with the time-causal limit kernel corresponds to first-order temporal integrators coupled in cascade, with very close relations to our understanding of temporal processing in neurons. Temporal derivatives can, in turn, be computed from linear combinations of temporally smoothed scale channels over multiple temporal scales, based on a similar recurrence relation over increasing orders of temporal differentiation as in Lindeberg and Fagerström (1996) [Equation (18)]. Thus, all the individual computational primitives needed to implement the spatial as well as the spatio-temporal receptive fields according to the generalised Gaussian derivative model can, in principle, be performed by computational mechanisms available to a network of biological neurons.

#### 3.2.1. Testable hypotheses from the theoretical predictions

For a visual neuron in the primary visual cortex that can be well modelled as a simple cell, let us assume that the spatio-temporal dependency of its receptive field can be modelled as a combination of a dependency on a spatial function *h*_*space*_(*x*_1_, *x*_2_) and a dependency on a temporal function *h*_*time*_(*t*) of the joint form^17^


(64) $$\begin{array}{l} h_{simple}\left(x_{1},x_{2},t\right)=h_{space}\left(x_{1}-v_{1}t,x_{2}-v_{2}t\right)h_{time}\left(t\right) \end{array}$$

for some value of a velocity vector *v* = (*v*_1_, *v*_2_), where we here in this model assume that both the spatial and the temporal dependency functions *h*_*space*_(*x*_1_, *x*_2_) and *h*_*time*_(*t*) as well as the velocity vector *v* are individual for each visual neuron. Then, we can formulate testable hypotheses for the above theoretical predictions in the following ways:

**Hypothesis 1** (Expansion of spatial receptive field shapes over a larger part of the affine group than mere rotations or uniform scale changes): Define characteristic lengths σ_φ_ and σ_⊥φ_ that measure the spatial extent of the spatial component *h*_*space*_(*x*_1_, *x*_2_) of the receptive field in the direction φ representing the orientation of the oriented simple cell as well as in its orthogonal direction ⊥φ, respectively. Then, if the hypothesis about an expansion over a larger part of the affine group than mere rotations is true, there should be a variability in the ratio of these characteristic lengths between different neurons


(65) $$\begin{array}{l} \epsilon =\frac{\sigma_{\varphi }}{\sigma_{⊥\varphi }}. \end{array}$$

If the spatial component of the receptive field *h*_*space*_(*x*_1_, *x*_2_) can additionally be well modelled as a directional derivative of an affine Gaussian kernel for some order of spatial differentiation, then this ratio between the characteristic lengths can be taken as the ratio between the effective scale parameters^18^ of the affine Gaussian kernel in the directions of the orientation of the receptive field and its orthogonal direction


(66) $$\begin{array}{l} \epsilon =\sqrt{\frac{s_{\varphi }}{s_{⊥\varphi }}}. \end{array}$$

**Hypothesis 2** (Joint expansion over image velocities and spatial eccentricities of the spatio-temporal receptive fields): Assuming that there is a variability in the ratio ϵ between the orthogonal characteristic lengths of the spatial components of the receptive fields between different neurons, as well as also a variability of the absolute velocity values


(67) $$\begin{array}{l} v_{speed}=\sqrt{v_{1}^{2}+v_{2}^{2}}, \end{array}$$

between different neurons, then these variabilities should together span a 2-D region in the composed 2-D parameter space, and not be restricted to only a set of 1-D subspaces, which could then be seen as different populations in a joint scatter diagram or a histogram over the characteristic length ratio ϵ and the absolute velocity value *v*_*speed*_.

**Hypothesis 3** (Separate expansions over image velocities and spatial eccentricities of the spatio-temporal receptive fields): The neurons that show a variability over the velocity values *v*_*speed*_ all have a ratio ϵ of the characteristic lengths in two orthogonal spatial directions for the purely spatial component of the receptive field that is close to constant, or confined within a narrow range.

Note that Hypotheses 2 and 3 are mutually exclusive.

#### 3.2.2. Quantitative characterizations of distributions of receptive field parameters

In addition to investigating if the above working hypotheses hold, which would then give more detailed insights into how the spatial and spatio-temporal receptive field shapes of simple cells are expanded over the degrees of freedom of the geometric image transformations, it would additionally be highly interesting to characterize the distributions of the corresponding receptive field parameters, in other words, the distributions of

- the spatial characteristic length ratio ϵ,
- the image velocity *v*_*speed*_,
- a typical spatial size parameter σ_*space*_, which for a spatial dependency function *h*_*space*_(*x*_1_, *x*_2_) in (13) corresponding to a directional derivative of an affine Gaussian kernel for a specific normalization of the spatial covariance matrix Σ would correspond to the square root of the spatial scale parameter, *i.e*., $\sigma_{space}=\sqrt{s}$, and
- a typical temporal duration parameter σ_*time*_, which for the temporal dependency function *h*_*time*_(*t*) in (13) corresponding to a temporal derivative of the time-causal limit kernel would correspond to the square root of the temporal scale parameter, *i.e*., $\sigma_{time}=\sqrt{\tau }$.

Characterising the distributions of these receptive field parameters, would give quantitative measures on how well the variabilities of the receptive field shapes of biological simple cells span the studied classes of natural image transformations, in terms of spatial scaling transformations, spatial affine transformations, Galilean transformations and temporal scaling transformations.

When performing a characterization of these distributions, as well as investigations of the above testable hypotheses, it should, however, be noted that special care may be needed, to only pool statistics over biological receptive fields that have a similar qualitative shape, in terms of the number of dominant positive and negative lobes over space and time. For the spatial and spatio-temporal receptive fields according to the studied generalised Gaussian derivative model, this would imply initially only collecting statistics for receptive fields that correspond to the same orders of spatial and temporal differentiation. Alternatively, if a unified model can be expressed for receptive fields of differing qualitative shape, as it would be possible if the biological receptive fields can be well modelled by the generalised Gaussian derivative model, statistics could also be pooled over different orders of spatial and/or temporal differentiation, by defining the characteristic spatial lengths from the spatial scale parameter *s* according to $\sigma_{space}=\sqrt{s}$, and the characteristic temporal durations from the temporal scale parameter τ according to $\sigma_{time}=\sqrt{\tau }$.

## 4. Summary and discussion

We have presented a generalised Gaussian derivative model for modelling visual receptive fields that can be modelled as linear, and which can be derived in an axiomatic principled manner from symmetry properties of the environment, in combination with structural constraints on the first stages of the visual system, to guarantee internally consistent visual representations over multiple spatial and temporal scales.

In a companion work (Lindeberg, 2021), it has been demonstrated that specific instances of receptive field models obtained within this general family of visual receptive fields do very well model properties of LGN neurons and simple cells in the primary visual cortex, as established in neurophysiological cell recordings by DeAngelis et al. (1995), DeAngelis and Anzai (2004), Conway and Livingstone (2006), Johnson et al. (2008). Indeed, based on the generalised Gaussian derivative model for visual receptive fields, it is possible to reproduce the qualitative shape of all the main types of receptive fields reported in these neurophysiological studies, including space-time separable neurons in the LGN, and simple cells in the primary visual cortex with oriented receptive fields having strong orientation preference over the spatial domain, as well as being either space-time separable over the joint spatio-temporal domain, or with preference to specific motion directions in space-time.

Specifically, we have in this paper focused on the transformation properties of the receptive fields in the generalised Gaussian derivative model under geometric image transformations, as modelled by local linearizations of the geometric transformations between single or multiple views of (possibly moving) objects or events in the world, expressed in terms of spatial scaling transformations, spatial affine transformations and Galilean transformations, as well as temporal scaling transformations. We have shown that the receptive fields in the generalised Gaussian derivative model possess true covariance properties under these classes of natural image transformations. The covariance properties do, in turn, imply that a vision system, based on populations of these receptive fields, will have the ability to, up to first order of approximation, handle: (i) the perspective mapping from objects or events in the world to the image or video domain, (ii) handle the image deformations induced by viewing image patterns of smooth surfaces in the world from multiple views, as well as (iii) the video patterns arising from viewing objects and events in the world, that move with different velocities relative to the observer, or (iv) spatio-temporal events that occur faster or slower in the world.

We argue that it is essential for a vision system to obey, alternatively sufficiently well approximate such covariance properties, in order to robustly be able to handle the huge variability of image of video data generated under the influence of natural image transformations. Based on covariant image and video measurements at early stages in the visual hierarchy, invariant representations can, in turn, be computed at higher levels. If the early stages in the visual system would not respect such basic covariance properties, or sufficiently good approximations thereof, the subsequent visual computations at higher levels would suffer from inherent measurement errors, caused by the non-infinitesimal extent and duration of the receptive fields over space and time, that may be otherwise hard to recover from. We do therefore argue that the covariance properties treated in this article are essential for both (i) the study and modelling of biological vision and (ii) the construction of artificial computer vision systems. Specifically, we argue that the influence of natural image transformations on the measurements of local image and video information based on visual receptive fields, is essential for understanding both the possibilities for and the computational functions in visual perception.

### 4.1. Relations to other sources of variability in image and video data

Beyond the variability due to natural geometric image transformations, which are handled by expanding the receptive field shapes over the degrees of freedom of the corresponding image transformations, it should be remarked that the presented model for visual receptive fields, does additionally have the ability to handle also other sources of variability in image and video data.

Concerning illumination variations, it can be shown that if receptive fields according to the generalised Gaussian derivative model for visual receptive fields, based on spatial and temporal derivatives of spatial and temporal smoothing kernels, are applied to image intensities expressed on a logarithmic brightness scale, then the receptive field responses will be automatically invariant to multiplicative intensity transformations, and thus be able to handle both (i) multiplicative changes in the illumination and (ii) multiplicative changes in exposure control mechanisms. In this way, a large source of variability regarding illumination changes is implicitly handled by the presented theory (Lindeberg, 2013, Section 2.3; Lindeberg, 2021, Section 3.4).

Concerning image and video noise, very fine scale receptive fields will have the property that they may respond primarily to fine scale surface textures and noise, whereas the noise and the fine scale textures will become effectively suppressed in coarser scale receptive fields. In this way, the coarser scale receptive fields will be more robust to image noise as well as the influence of very fine scale surface textures.

Concerning transparencies, an interesting property of a multi-parameter model for receptive fields, as considered here, is also that it can respond to qualitatively different types of image structures for different values of the receptive field parameters. Beyond different types of responses at different scales, which as previously considered can handle spatial structures at different scales, by varying the velocity parameter *v* in the spatio-temporal receptive field model in Equation (9), such a model will have the ability to handle aspects of transparent motion, in the sense that by considering the receptive field responses for the parameters of Galilean motion that describe the motion of foreground image structures *vs*. the background image structure in a two-layer transparent motion. By extending the receptive field model to binocular receptive fields over a disparity parameter, obtained by varying the parameter δ_(*x*_1_, *x*_2_)_ in Equation (1), such an extended model of visual receptive fields would also have the ability to handle static transparencies.

In these ways, the multi-parameter model of receptive fields considered here can also serve other purposes, beyond handling geometric image transformations, as a basis for early vision. If aiming at extending the model to other sources of variability, such as handling occlusions, then a natural starting point to use is to start from the generalised diffusion equations (1) and (8) that generate the corresponding visual receptive fields, and then complement with explicit learning mechanisms over the corresponding parameters in the receptive fields, and also to, for example, complement with explicit end-stopping mechanisms to prevent the smoothing process from extending over object boundaries.

### 4.2. Relations to previous work

In their ground-breaking work, (Hubel and Wiesel, 1959, 1962, 1968, 2005) characterised properties of the receptive fields for simple and complex cells in the primary visual cortex (V1). In their experimental methodology, they used moving light bars that made it possible to capture qualitative properties of receptive fields, such as the orientation selectivity in V1. Later studies based on more refined stimuli, such as white-noise-patterns, have then made it possible to reconstruct more detailed characterizations of biological visual receptive fields from multiple measurements of the same cell (DeAngelis et al., 1995; Ringach, 2002, 2004; DeAngelis and Anzai, 2004; Conway and Livingstone, 2006; Johnson et al., 2008; Ghodrati et al., 2017; De and Horwitz, 2021). Summarising these results in a qualitative manner, it has been found that a majority of the receptive fields in the retina and the LGN are rotationally symmetric over the spatial domain and space-time separable over the spatio-temporal domain, whereas simple cells in V1 have strong orientation preference over the spatial domain, as well as that the simple cells are either space-time separable over the spatio-temporal domain or tuned to particular motion directions in joint space-time.

Learning-based schemes, which learn receptive fields from collections of training data, have been formulated, trying to explain those types of receptive fields found in biologically vision. Rao and Ballard (1998) demonstrated how localised oriented receptive fields could be obtained by learning a translation-invariant code for natural images. Olshausen and Field (1996, 1997) proposed that properties of receptive fields similar to biological receptive fields could be obtained by learning a sparse code for natural images. Simoncelli and Olshausen (2001), Geisler (2008), Hyvärinen et al. (2009) argued that the properties of neural representations are determined by natural image statistics. Lörincz et al. (2012) proposed that early sensory processing can be modelled by sparse coding. Poggio and Anselmi (2016) proposed to model learning of invariant receptive fields by using group theory. Singer et al. (2018) used the proxy task of predicting the relative future in pre-recorded video sequences for training a deep network, and demonstrated how that approach lead to receptive field shapes with good qualitative similarities to biological receptive fields. Deep neural network approaches for analysing and modelling non-linear receptive fields of sensory neural responses have also been developed (Keshishian et al., 2020); see also the more general discussions concerning such methodologies in Bae et al. (2021), Bowers et al. (2022), Heinke et al. (2022), Wichmann and Geirhos (2023) and the references therein.

Mathematically based computational models have also been formulated to reflect the shapes of receptive fields found in biological vision. Rodieck (1965) proposed to model circularly symmetric receptive fields in the retina and the lateral geniculate nucleus (LGN) by differences-of-Gaussians. Marcelja (1980) as well as Jones and Palmer (1987a,b) proposed to model simple cells by Gabor functions, motivated by their property of minimising the uncertainty relation; see also Porat and Zeevi (1988) for a more general proposal of using the Gabor filter model for visual operations. Riesenhuber and Poggio (1999) built on these ideas, and used Gabor functions in a hierarchical model of object recognition. Young (1987), Young and Lesperance (2001), Young et al. (2001) proposed to instead model simple cells by Gaussian derivatives, with close relations to theoretical arguments in support for the (regular) Gaussian derivative model stated by Koenderink (1984), Koenderink and van Doorn (1987, 1992). More detailed models of biological receptive fields based on the associated (regular) Gaussian derivative framework have, in turn, been presented by Lowe (2000), Hesse and Georgeson (2005), Georgeson et al. (2007), May and Georgeson (2007), Hansen and Neumann (2008), Wallis and Georgeson (2009), Pei et al. (2016), Wang and Spratling (2016).

The generalised Gaussian derivative theory for visual receptive fields, that we have built upon in this work, can mathematically derive receptive field shapes directly from symmetry properties of the environment in an axiomatic manner (Lindeberg, 2011, 2013, 2016, 2021) regarding a first layer of linear receptive fields, and can well model biological receptive fields in the retina, the lateral geniculate nucleus and the primary visual cortex. A conceptual similarity between this theoretical approach and the above learning-based approaches is that the structural properties of the environment will imply strong constraints on the statistics of natural images, and thus the properties of the training data that the receptive field shapes are learned from. Starting directly from the symmetry properties of the world, thereby shortcircuits the need for learning receptive field shapes from collections of training data, provided that the mathematical analysis from the structural assumptions to the receptive field shapes can be tractable.

### 4.3. Extensions to non-linear visual receptive fields and artificial deep networks

While the presented theory can be seen as theoretically rather complete, as a model for the earliest layers of linear receptive fields in an artificial vision system, or for the biological receptive fields in the retina, the lateral geniculate nucleus and the primary visual cortex, including their relations to the influence of geometric image transformations, an interesting problem concerns how to extend the theory to higher layers in the visual hierarchy, as well as to non-linear image and video operations. Regarding the specific problem of achieving spatial scale covariance in a non-linear hierarchy of visual receptive fields, a general theoretical sufficiency result was presented in Lindeberg (2020), which guarantees spatial scale covariance for a hierarchical vision model based on a set of homogeneous non-linear polynomial or rational combinations of scale-normalised Gaussian derivatives coupled in cascade, including pointwise self-similar transformations thereof. For a specific biomimetic implementation of this general idea, in terms of an oriented quasi quadrature model that reproduces some of the known qualitative properties of complex cells, a scale-covariant hierarchical network architecture was formulated, with close conceptual similarities to the scattering network formulation of deep networks proposed by Mallat (2016). Specifically, it was demonstrated that, due to the scale invariant properties that arise from the resulting provably scale-covariant network, it was possible to perform predictions over spatial scales, to perform training at one scale and testing at other scales, not spanned by the training data (see Figures 15 and 16 in Lindeberg, 2020).

More developed approaches to such scale generalization based on scale-covariant and scale-invariant deep networks were then presented in Jansson and Lindeberg (2022), Lindeberg (2022). The approach in Lindeberg (2022) is based on coupling linear combinations of scale-normalised Gaussian derivatives in cascade, with pointwise non-linearities between, with close similarity to the previous work on using Gaussian derivative kernels as structured receptive field models in deep networks by Jacobsen et al. (2016); see also Pintea et al. (2021), Penaud et al. (2022) for parallel work on using Gaussian derivatives as primitive filters in deep networks. The approach in Jansson and Lindeberg (2022) is instead based on building a deep network with multiple spatial scale channels, defined by applying the same discrete deep network to multiple rescaled copies of the input image, thus achieving scale-covariant and scale-invariant properties in a dual manner, by performing multiple rescalings of the input image, as opposed to applying multiple spatially rescaled spatial receptive fields to the same input image, and leading to very good scale generalization properties in experiments. It was also demonstrated how the resulting scale-invariant multi-scale network was able to learn more efficiently from sparse training data, compared to a single-scale network, in that the multiple spatial scale channels could support each other in the training phase, and make more efficient use of multi-scale training data than a regular single-scale network. Sangalli et al. (2022), Yang et al. (2023) have performed closely related work on scale generalization based on scale-covariant U-Nets.

More generally, Worrall and Welling (2019), Bekkers (2020), Sosnovik et al. (2020, 2021a,b), Zhu et al. (2022) have developed scale-covariant or scale-equivariant deep network architectures, and demonstrated that these lead to more robust results under variations in the scale of image structures, compared to non-covariant or non-equivariant counterparts. (Barisin et al., 2023) have developed related methods for handling multi-scale image structures and performing scale generalization based on scale-invariant Riesz networks.

Currently, there is an active area of research to develop covariant or equivariant deep networks, where we propose that it should be natural to consider generalizations of the covariance properties under natural image transformations treated for the receptive field models in this article. We do also more generally propose to include more explicit treatments of the influence of natural image transformations, such as covariance and invariance properties under the classes of geometric image and video transformations studied in this article, in both the study as well as the computational modelling of biological vision.

## Data availability statement

The original contributions presented in the study are included in the article/Supplementary material, further inquiries can be directed to the corresponding author.

## Author contributions

TL developed the theory, generated the illustrations, and wrote the article.

## Footnotes

^1^Whereas the notion of a visual receptive field traditionally refers to the region in the visual field over which a neuron responds to visual stimuli (Hartline, 1938), we here complement that definition to also comprise the computational function of the neuron over the part in the visual field in which it reacts to visual patterns.

^2^In the deep learning literature, the property that we refer to as “covariance” is often referred to as “equivariance.” In this paper, we use the term “covariance” because of the traditional use of this terminology in physics, and to maintain consistency with the previous work in scale-space theory that this paper builds upon. An operator *O* is said to be covariant under a transformation group *T*_*p*_ with parameter *p*, if the operator essentially commutes with the transformation group, in the sense that *O*′(*T*_*p*_*(f*)) = *T*_*p*_(*Of*) for some possibly transformed operator *O*′ within the same family of operators as *O*.

^3^A semi-group structure over convolutions with a family of kernels *T*(*x*_1_, *x*_2_; *s*), where *s* is the parameter of the semi-group, means that the result of convolving two kernels with each other will be a kernel of the same family under addition of the parameters, *i.e*., *T*(·, ·;*s*_1_)**T*(·, ·;*s*_2_) = *T*(·, ·;*s*_1_ + *s*_2_). Note that a convolution structure is implied from the previous assumptions of linearity and translation covariance.

^4^Non-enhancement of local extrema means that the value of the image representation at any local maximum over the image domain must not increase from finer to coarser levels of scales, and that the value of the image representation at any local minimum over the image domain must not decrease.

^5^In the model below, the parameters *s* and Σ are not independent, implying that we could multiply the spatial scale parameter *s* with some constant *C*, while dividing the spatial covariance matrix Σ with the same constant, and still get the same spatial receptive field. To handle this problem, we can introduce a convention to normalize Σ in some way, for example, by choosing a normalization such that the maximum eigenvalue of Σ is equal to 1. For a monocular view of smooth surface under orthographic projection, such a normalization is closely related to the foreshortening transformation from the tangent plane of the surface to the image plane. If the slant angle of the surface is σ relative to the viewing direction, then a rotationally symmetric receptive field, when backprojected to the tangent plane of the surface, would correspond to the minimum eigenvalue of Σ being cos^2^σ.

^6^It is, however, possible to complement the spatio-temporal LGN model (16) by velocity-adaptation, leading to a receptive field model of the form $h_{LGN}\left(x_{1},x_{2},t;s,\tau ,v\right)=\pm s\tau^{n/2}\left(\partial_{x_{1}x_{1}}+\partial_{x_{2}x_{2}}\right)\partial_{{\bar{t}}^{n}}\left(g\left(x-v_{1}t,y-v_{2}t;s\right)\psi \left(t;\tau ,c\right)\right)$, to also obtain Galilean covariance. Notably, there are neurophysiological results indicating that there also exist neurons in the LGN that are sensitive to motion directions (Ghodrati et al., 2017, Section 1.4.2).

^7^In the area of scale-space theory, these spatially smoothed image representations are referred to as (here affine) spatial scale-space representations.

^8^In the area of scale-space theory, these spatio-temporally smoothed video representations are referred to as spatio-temporal scale-space representations.

^9^The reason why the temporal scaling factor is here restricted to being an integer power of the distribution parameter of the time-causal limit kernel, is that the temporal scale levels resulting from convolution with this temporal smoothing kernel are genuinely discrete. Thereby, exact temporal scale covariance can only be obtained for temporal scaling factors that perfectly match the available ratios between the temporal scale levels in the corresponding temporal multi-scale representation. For other scaling factors, the results will instead only be numerical approximations, whose accuracy depends on the density of the discrete temporal scale levels, as determined by the distribution parameter *c* > 1 of the time-causal limit kernel.

^10^If the time-causal limit kernel in the general form (9) of spatio-temporal smoothing kernels, that this treatment is based on, is replaced by a non-causal temporal Gaussian kernel, then a corresponding temporal scale covariance will also hold, however then instead over a continuum of temporal scaling factors *S*_*t*_. as opposed to a discrete subset as for the time-causal model.

^11^The view followed in this treatment, that the primary visual cortex performs a substantial expansion of the image and video data over the parameters of the geometric image and video transformations, is consistent with the substantial expansion of measurement data that is performed from the LGN, with about 1 M neurons and 1 M output channels, to V1, with 190 M neurons and 37 M output channels, see DiCarlo et al. (2012) (Figure 3).

^12^And taking the square root of the ratio between the eigenvalues of the spatial covariance matrix Σ, to base the eccentricity measurement on measurements in unit of [length], as opposed to units of [length^2^]. Geometrically, variations in this ratio between the square root of the ratio between the eigenvalue does, for example, make it possible to handle variations in the slant angle of a local surface patch viewed under monocular projection.

^13^In relation to the fact that the measurements of orientation selectivity by Nauhaus et al. (2008) may most likely involve complex cells, whereas the predictions in this theory are based on the response properties of simple cells, it can be noted that that this qualitative result concerning the orientation selectivity of simple cells, based on the affine Gaussian derivative model, will also extend to complex cells, if the complex cells can be modelled as operating on the output of simple cells as done in Lindeberg (2020), Section 5, see Lindeberg (2023a) for a detailed analysis of this subject.

^14^We implicitly regard variability over image velocities as established, since velocity-tuned receptive fields have been recorded for different image velocities.

^15^The semi-group property over spatial scales *s* of the rotationally symmetric Gaussian kernel *g*(*x*; *s*) implies that the convolution of two rotationally symmetric Gaussian kernels with each other is also a rotationally symmetric Gaussian kernel, and with added scale parameters *g*(*x*; *s*_1_)**g*(*x*; *s*_2_) = *g*(*x*; *s*_1_ + *s*_2_), whereas the semi-group property over the spatial covariance matrices Σ of the affine Gaussian kernel means that the convolution of two affine Gaussian kernels is also an affine Gaussian kernel, and with added spatial covariance matrices *g*(*x*; Σ_1_)**g*(*x*; Σ_2_) = *g*(*x*; Σ_1_ + Σ_2_). These semi-group properties do, in turn, mean that spatial smoothed representations with the rotationally symmetric Gaussian kernel are related according to the spatial cascade smoothing property *L*(*x*; *s*_2_) = *g*(*x*; *s*_2_ − *s*_1_)**L*(*x*; *s*_1_), provided that *s*_2_ > *s*_1_, whereas spatially smoothed representations with the affine Gaussian kernel are related according to spatial cascade smoothing property *L*(*x*; Σ_2_) = *g*(*x*; Σ_2_ − Σ_1_)**L*(*x*; Σ_1_), provided that Σ_2_ − Σ_1_ is a symmetric positive definite matrix. With regard to the spatial component of the model for the receptive fields of LGN neurons (7), the spatial cascade smoothing property originating from the semi-group property of the rotationally symmetric Gaussian kernel implies that the output from the spatial LGN model (7) at spatial scale *s*_2_ is related to the corresponding output at spatial scale *s*_1_ according to $L_{LGN}\left(x;s_{2}\right)=s^{s_{2}-s_{1}}g\left(x;s_{2}-s_{1}\right)*L_{LGN}\left(x;s_{1}\right)$, provided that *s*_2_ > *s*_1_, whereas the output from the spatial model of the receptive fields of simple cells (6) at spatial scale *s*_2_ is related to the corresponding output at spatial scale *s*_1_ according to $L_{simple}\left(x;s_{2},\Sigma \right)=s^{\left(s_{2}-s_{1}\right)m/2}g\left(x;\left(s_{2}-s_{1}\right)\Sigma \right)*L_{simple}\left(x;s_{1},\Sigma \right)$, provided that *s*_2_ > *s*_1_ and that the direction of the directional derivative operator in this model for simple cells is aligned to the eigenvectors of the spatial covariance matrix Σ.

^16^Due to the cascade smoothing property of the time-causal limit kernel over temporal scales, the time-causal limit kernels Ψ(*t*; τ, *c*) at adjacent temporal scales are related according to $\text{Ψ}\left(t;\tau ,c\right)=h_{exp}\left(t;\frac{\sqrt{c^{2}-1}}{c}\sqrt{\tau }\right)*\text{Ψ}\left(t;\frac{\tau }{c^{2}},c\right)$, with *h*_*exp*_(*t*; μ) denoting a truncated exponential kernel with time constant μ according to $h_{exp}\left(t;\mu \right)=\frac{1}{\mu_{k}}e^{-t/\mu_{k}}$ and *c* being the distribution parameter of the time-causal limit kernel [Lindeberg, 2023b, Equation (28)]. This does, in turn, mean that temporal derivatives of purely temporally smoothed representations at adjacent temporal scales are related according to $L_{t^{n}}\left(t;\tau ,c\right)=h_{exp}\left(t;\frac{\sqrt{c^{2}-1}}{c}\sqrt{\tau }\right)*L_{t^{n}}\left(t;\frac{\tau }{c^{2}},c\right)$.

^17^This model is inspired by the theoretically derived spatio-temporal receptive field model (13), however, generalised to more spatial and temporal dependency functions than directional derivatives of affine Gaussian kernels or temporal derivatives of the time-causal limit kernel, and with the explicit dependencies on the transformation parameters now included in the separate spatial and temporal dependency functions for an individual neuron. The model is also inspired by biological results concerning a preference of biological receptive fields to specific motion directions space-time.

^18^Note that the directional derivative of an affine Gaussian kernel will be separable in a coordinate system aligned to the orientation of the receptive field, provided that the direction for computing the directional derivative is aligned with one of the eigendirections of the covariance matrix Σ associated with the affine Gaussian kernel.
