# Erratum: Covariance properties under natural image transformations for the generalised Gaussian derivative model for visual receptive fields

**DOI:** 10.3389/fncom.2023.1282093

**Published:** 2023-09-01

**Authors:** Frontiers Production Office

**Affiliations:** Frontiers Media SA, Lausanne, Switzerland

**Keywords:** receptive field, Image transformations, scale covariance, affine covariance, Galilean covariance, primary visual cortex, vision, theoretical neuroscience

Due to a production error, there was a mistake in Footnote 2 in the HTML version of the article. A correction has been made to the mathematical expression. The corrected footnote appears below.

2. In the deep learning literature, the property that we refer to as “covariance” is often referred to as “equivariance.” In this paper, we use the term “covariance” because of the traditional use of this terminology in physics, and to maintain consistency with the previous work in scale-space theory that this paper builds upon. An operator *O* is said to be covariant under a transformation group *T*_*p*_ with parameter *p*, if the operator essentially commutes with the transformation group, in the sense that *O*'(*T*_*p*_*(f*)) = *T*_*p*_(*Of*) for some possibly transformed operator *O*' within the same family of operators as *O*.

The publisher apologizes for this mistake. The original article has been updated.

